# Hac1-independent functions of Ire1 drive morphogenesis in *Candida albicans* through distinct transcriptional programs

**DOI:** 10.1242/jcs.264610

**Published:** 2026-07-21

**Authors:** Samuel Stack-Couture, Gabriela Nunes Marsiglio Librais, Bryan Lung, Julie Genereaux, Viola Halder, Vanessa Dumeaux, Rebecca S. Shapiro, Patrick Lajoie

**Affiliations:** ^1^ Department of Anatomy and Cell Biology, The University of Western Ontario, London, Ontario N6A 5C1, Canada; ^2^ Department of Molecular and Cellular Biology, University of Guelph, Guelph, Ontario N1G 2W1, Canada; ^3^ Department of Biochemistry, The University of Western Ontario, London, Ontario N6A 5C1, Canada; ^4^ Department of Oncology, Lawson Research Institute, London Health Sciences Centre, London, Ontario N6A 4V2, Canada; ^5^ Children's Health Research Institute, Lawson Health Research Institute, London, Ontario N6C 2V5, Canada

**Keywords:** UPR, *Candida albicans*, Endoplasmic reticulum, Morphogenesis

## Abstract

The pathogenic yeast *Candida albicans* relies on morphogenesis – the transition from spherical yeast to filamentous hyphal forms – for infection. Although morphogenesis requires Ire1, a transmembrane protein that canonically initiates the unfolded protein response (UPR) through *HAC1* mRNA splicing, the specific mechanisms linking Ire1 to filamentation remain unclear. Using transcriptome analysis, we found that the Ire1-dependent transcriptional response driving morphogenesis was fundamentally distinct from the canonical UPR response to proteotoxic stress, with minimal overlap between programs. Morphogenesis was associated with only limited *HAC1* splicing compared to the robust splicing seen during proteotoxic stress, and *HAC1* deletion only partially impaired filamentation, unlike the near-complete loss observed with *IRE1* deletion. These findings establish that Ire1 regulates hyphal development through previously uncharacterized *HAC1*-independent pathways. We identify cell wall integrity as a key *HAC1*-independent mechanism, with Ire1 – but not Hac1 – being essential for cell wall stress tolerance and upregulation of cell wall biosynthesis genes during filamentation. Our data also reveal Ire1-dependent decreases in transcripts encoding secretory proteins during both proteotoxic stress and morphogenesis, consistent with a possible role for Ire1-mediated mRNA degradation in these processes. Given the essential role of Ire1 in pathogenesis and extensive development of Ire1-targeting compounds for mammalian systems, our findings position Ire1 as a highly promising druggable target for novel antifungal therapeutics and development of fungal-specific inhibitors.

## Teaser

**Summary:** Hyphal development in *Candida albicans* requires Ire1 but only minimally engages Hac1 splicing, revealing distinct Hac1-independent transcriptional programs that make Ire1 a compelling antifungal therapeutic target.

## INTRODUCTION

Fungal disease is highly prevalent throughout the human population and causes an estimated 1.6 million deaths per year (Bongomin et al., 2017). *Candida* species are one of the most common agents to cause fungal infections, with *Candida albicans* being the most common cause of invasive *Candida* infections, or candidiasis (Pappas et al., 2018; Shapiro et al., 2011; Soriano et al., 2023; Talapko et al., 2021). *Candida* infections can be life threatening to immunocompromised individuals living with HIV or AIDS (Anwar et al., 2012; Keyvanfar et al., 2024; Li et al., 2013; Patil et al., 2018), individuals undergoing chemotherapy (Matsuo et al., 2024; Sharifi et al., 2023; Teoh and Pavelka, 2016) and transplant recipients (Carugati et al., 2024; Senoner et al., 2023; Seok et al., 2020; Shoham and Marr, 2012).

*C. albicans* infection largely relies on the ability of this yeast to undergo morphogenic changes from the spherical yeast form to filamentous pseudohyphal and true hyphal forms (Brunke and Hube, 2013; Mayer et al., 2013). The yeast form is important for dissemination through the bloodstream, whereas the filamentous forms enable *C. albicans* to escape phagocytic immune cells and penetrate host tissues (Berman, 2006; Mayer et al., 2013). Filamentation can be induced experimentally through various mechanisms including serum treatment, elevated temperature, nutrient starvation, chromatin accessibility and pH neutralization (Do et al., 2025; Jenull et al., 2017; Razzaq et al., 2021; Shapiro et al., 2011; Shivarathri et al., 2019; Tebbji et al., 2017; Villa et al., 2020). Changes in proteostasis also affect morphogenesis; Hsp90 inhibition, *HSF1* overexpression or depletion and proteasome inhibition all regulate *C. albicans* filamentation (Hossain et al., 2020; Shapiro et al., 2009; Veri et al., 2018). Transcriptional control of morphogenesis is mediated by multiple pathways including the cAMP-PKA and mitogen-activated protein kinase (MAPK) pathways, and a pH-dependent pathway dependent on the transcription factor Rim101 (Villa et al., 2020). The cAMP-PKA pathway, mediated by both the ammonium permease Mep2 and the G-protein-coupled receptor (GPCR) Grp1, is activated in response to nitrogen and amino acid starvation, high temperature and increased carbon dioxide (Huang et al., 2019; Shapiro et al., 2011). cAMP-PKA activation leads to upregulation of Efg1, a key transcription factor involved in filamentation (Braun and Johnson, 2000; Glazier, 2022). Mep2 also activates the MAPK pathway, which leads to transcriptional regulation of filamentation-specific genes by Cph1 (Shapiro et al., 2011). Morphogenesis is also negatively regulated by transcription factors Nrg1 and Tup1 (Braun and Johnson, 1997, 2000; Braun et al., 2000, 2001).

Morphogenesis in *C. albicans* requires the kinase inositol-requiring enzyme 1 (Ire1) (Blankenship et al., 2010; Sircaik et al., 2021; Zhao et al., 2025), a transmembrane protein responsible for activating the unfolded protein response (UPR) in yeast (Bashir et al., 2021; Cox et al., 1993; Mori et al., 1993; Nikawa and Yamashita, 1992). The UPR is a highly conserved signaling pathway first characterized in the budding yeast *Saccharomyces cerevisiae*. Upon activation by unfolded proteins in the endoplasmic reticulum (a condition termed ER stress) or lipid bilayer stress (Covino et al., 2018; Ho et al., 2020; Lajoie et al., 2012; Promlek et al., 2011), Ire1 facilitates the splicing of an intron from *HAC1* mRNA (Cox and Walter, 1996; Mori et al., 1996). This results in the synthesis of the Hac1 transcription factor (Geronimo et al., 2025; Matabishi-Bibi et al., 2022; Sato et al., 2025), which translocates to the nucleus to upregulate hundreds of genes involved in processes such as ER-associated degradation, chaperone-mediated protein folding and lipid biosynthesis, reflecting the breadth of the cellular functions regulated by the UPR (Thibault et al., 2011; Travers et al., 2000; Walter and Ron, 2011).

In several species of plants, animals and fungi, Ire1 cleaves ER-localized mRNAs and targets them for degradation through a mechanism called regulated Ire1-dependent decay (RIDD) (Han et al., 2009; Hernández-Elvira et al., 2018; Hollien and Weissman, 2006; Hollien et al., 2009). In *Schizosaccharomyces pombe*, there is no *HAC1* ortholog or transcriptional response to ER stress, and ER stress is instead alleviated by reducing the translation burden of the cell through RIDD (Guydosh et al., 2017; Kimmig et al., 2012). Similarly, the pathogenic yeast *Candida glabrata* mediates ER stress in an Ire1-dependent Hac1-independent manner through RIDD. *C. glabrata* does possess a *HAC1* ortholog, although *HAC1* mRNA is not spliced in response to ER stress conditions and the transcriptional response to ER stress appears to depend more on the calcineurin-Crz1 pathway (Miyazaki et al., 2013). The *C. albicans* ER stress response is canonically mediated by the Hac1 transcriptional response. It is unclear whether *C. albicans* possesses RIDD; however, there are several known Hac1-independent functions of Ire1 in *C. albicans*. Ire1 is required for iron uptake and virulence independent of Hac1 (Ramírez-Zavala et al., 2022), and Ire1 attenuates ER stress by activating the HOG-MAPK pathway in a manner that might not involve Hac1 (Husain et al., 2021).

Loss of Ire1 function reduces *C. albicans* filamentation and biofilm formation, leading to decreased virulence in mouse models of disseminated candidiasis (Blankenship et al., 2010; Ramírez-Zavala et al., 2022; Sircaik et al., 2021). Although Ire1 is known to be required for pathogenicity in *C. albicans*, the specific target genes downstream of Ire1 involved in this process remain uncharacterized. Additionally, deleting the transcription factor Hac1 in *C. albicans* was initially thought to result in a strain that is unable to undergo filamentation (Wimalasena et al., 2008); however, a recent study has demonstrated that Hac1 is only partially required for *C. albicans* filamentation and suggested that Hac1-independent functions of Ire1 are also required for morphogenesis (Lee et al., 2023). Although these studies have established that Ire1 and Hac1 have separable roles, the specific Ire1-dependent transcriptional programs that drive morphogenesis, and whether they overlap with canonical UPR targets, have not been defined at a genome-wide level. Here, we performed comprehensive transcriptome analysis to characterize the specific Ire1-mediated transcriptional response required for *C. albicans* morphogenesis. We also sought to define the involvement of Hac1 in *C. albicans* morphogenesis and characterize Hac1-independent functions of Ire1 in the process.

## RESULTS

### *IRE1* is required for morphogenesis in *C. albicans*

**Fig. 1.**
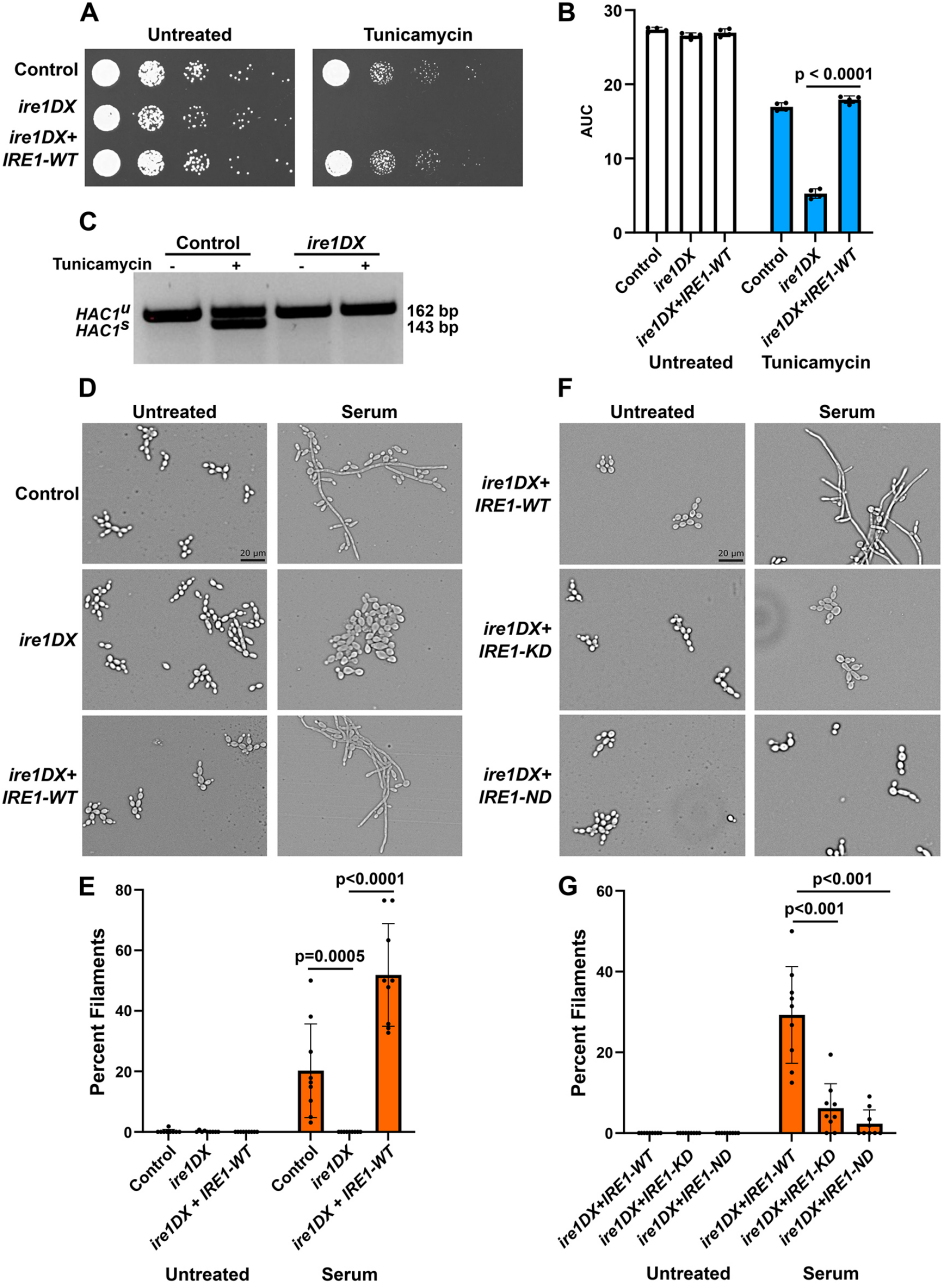
***IRE1* is required for the *C. albicans* response to the ER stressor tunicamycin and for morphogenesis.** (A) Control (DAY286), *ire1DX* and *ire1DX+IRE1-WT C. albicans* cells were diluted to an OD_600_ of 0.1, serially diluted and grown on agar plates with the canonical ER stressor tunicamycin (1.5 μg/ml). Images were acquired after 24 h. Images representative of *n*=3. (B) Cells were treated with 2 μg/ml tunicamycin and grown in liquid medium for 24 h. The area under the curve (AUC) was calculated and statistical analysis performed using a one-way ANOVA with Tukey's multiple comparisons test (*n*=4). Results are mean±s.d. (C) Cells were treated with 2.5 μg/ml tunicamycin for 2 h and total RNA was isolated and purified. RT-PCR was performed using primers flanking the *HAC1* intron and RT-PCR products were resolved on a 4% agarose gel. Images representative of three repeats. u, unspliced form; s, spliced form. (D,F) The indicated *C. albicans* strains were grown in filamentation-inducing 10% fetal bovine serum for 4 h at 37°C and untreated at 30°C as a control. Images obtained using the BioTek Cytation 5 Cell Imaging Multimode Reader. Scale bar: 20 μm, applies to all images. KD, kinase dead; ND, nuclease dead. (E,G) Filamentation was quantified for three biological replicates. Photos were acquired from randomly selected portions of each slide, and round and filamentous cells were classified and counted by hand for each photo. Percentage filaments was calculated and mean±s.d. was plotted. Statistical analysis performed using one-way ANOVA with Tukey's multiple comparisons.

To study the unfolded protein response, we used a strain with diminished expression (denoted DX) of *IRE1* (*ire1DX*). The *ire1DX* strain contains a deletion of one *IRE1* allele with the second *IRE1* allele placed under control of the weakly expressed *PGA5* promoter (Woolford et al., 2016). A complemented *ire1DX+IRE1-WT* strain was generated as previously described to serve as a control (Sircaik et al., 2021). The *ire1DX* strain was hypersensitive to tunicamycin (Fig. 1A,B), an N-linked glycosylation inhibitor that causes ER stress (Kuo and Lampen, 1974). *ire1DX* was also unable to undergo *HAC1* splicing in the presence of tunicamycin, confirming that it has dysfunctional UPR activation (Fig. 1C). To confirm the requirement of Ire1 in *C. albicans* morphogenesis, we performed a filamentous growth assay and found that *ire1DX* was unable to undergo filamentation when grown at 37°C in 10% fetal bovine serum (FBS) (0% filaments; Fig. 1D). In contrast, the parental DAY286 strain and *ire1DX+IRE1-WT* control had 20.2% and 51.9% filaments, respectively, consistent with previous findings (Fig. 1E; Blankenship et al., 2010; Sircaik et al., 2021). Next, we examined whether the kinase and nuclease domains of Ire1 are necessary for morphogenesis by complementing the *ire1DX* strain with a previously described ‘kinase-dead’ (KD) or ‘nuclease-dead’ (ND) copy of *IRE1* (Sircaik et al., 2021). We found that both the kinase and nuclease domains of Ire1 were required for morphogenesis, as demonstrated by a 23% and a 27% decrease in mean percentage of cells undergoing filamentous growth (denoted percentage filaments) relative to the control, respectively (Fig. 1F,G). Both functions of Ire1 were also required for *C. albicans* growth on tunicamycin (Fig. S2) (Sircaik et al., 2021). Notably, despite the significant evolutionary divergence between these species, *C. albicans IRE1* was able to functionally replace *S. cerevisiae IRE1* (Fig. S3A,B), indicating that the *HAC1* splicing capability has been evolutionarily maintained.

### A distinct *IRE1* transcriptional response drives morphogenesis

**Fig. 2.**
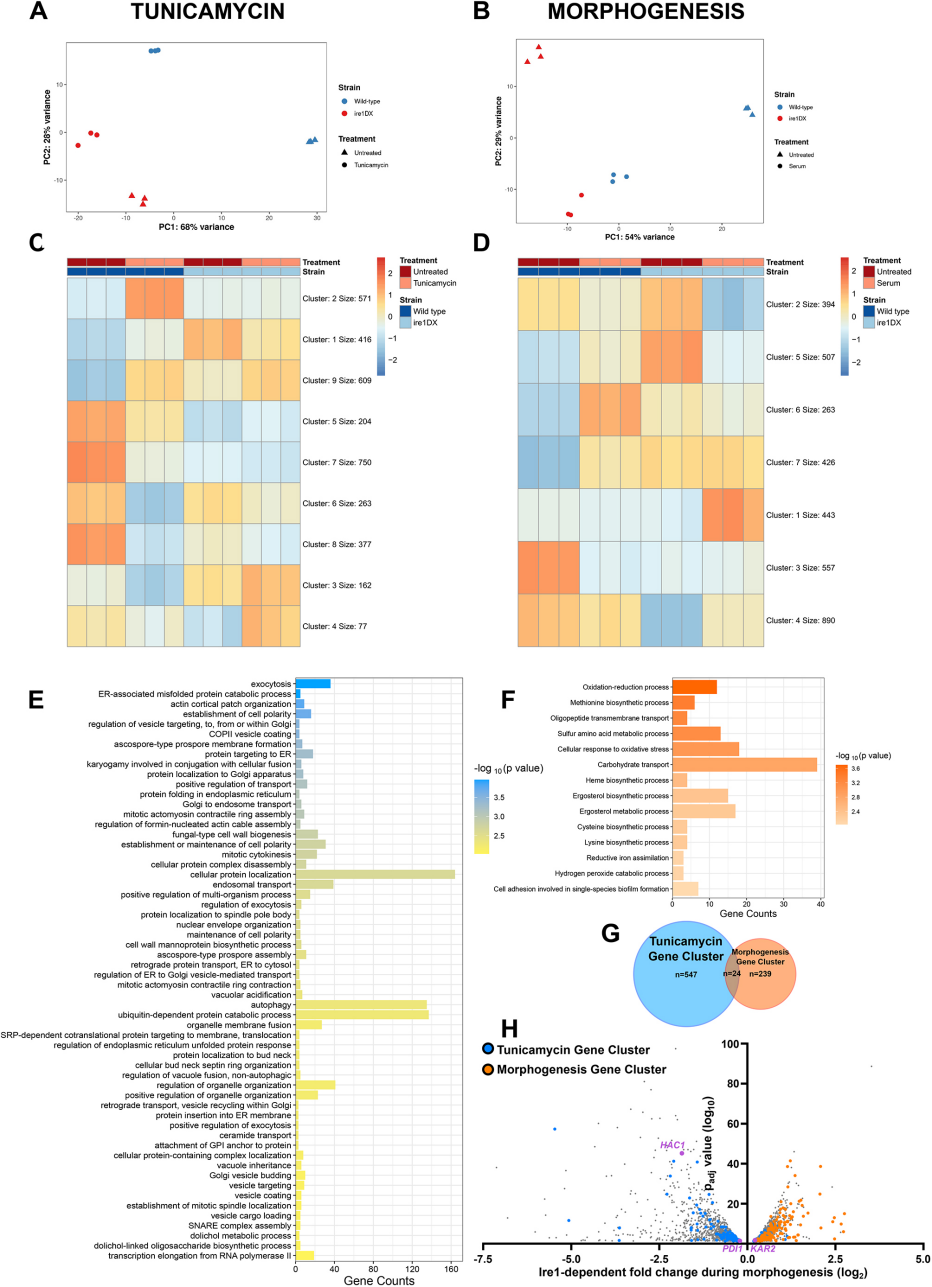
**Clustering of differentially expressed genes reveals genes upregulated by Ire1 in response to tunicamycin and serum+37°C treatment.** (A,B) Principal component analysis of control (DAY286) and *ire1DX* treated with (A) 1.5 μg/ml tunicamycin or (B) 10% fetal bovine serum+37°C. Each point represents a single biological replicate (*n*=3). (C,D) RNA sequencing data from control (DAY286) and *ire1DX* treated with (C) tunicamycin or (D) serum+37°C were analyzed. Differentially expressed genes with a significant treatment×strain interaction (*P*_adj_<0.05) were clustered into groups based on similar expression profiles (*n*=3429 genes for tunicamycin and n=3480 genes for morphogenesis). (E,F) Gene ontology analysis for biological processes was performed using ViSEAGO in R Studio (Brionne et al., 2019) for (E) tunicamycin cluster 2 and (F) morphogenesis cluster 6. Only terms with *P*<0.01 are displayed. (G) Venn diagram representing total number of genes in tunicamycin cluster 2 and morphogenesis cluster 6, showing there is little overlap. (H) Volcano plot of differentially expressed genes (*P*_adj_<0.05). Fold change and *P*_adj_ values calculated from the Ire1-dependent fold change for each gene in serum [log_2_(WT serum+37°C/*ire1DX* serum+37°C)]. Blue dots represent genes in tunicamycin cluster 2. Orange dots represent genes in serum cluster 6. Grey dots represent genes not in either cluster. Purple dots represent UPR genes of interest.

Given the requirement for Ire1 during morphogenesis, we sought to determine the Ire1-regulated target genes involved in morphogenesis. Wild-type cells and *ire1DX* were treated with serum and grown at 37°C for 4 h and processed for transcriptome analysis using RNA-seq. To compare the transcriptional response associated with filamentous growth to a canonical Ire1-mediated proteotoxic stress response, wild-type and *ire1DX* cells were treated with tunicamycin for 2 h and similarly processed. Dimension reduction by principal component analysis showed that biological replicates for the same condition clustered close together and highlighted a treatment effect that is dependent on strain (Fig. 2A,B).

To identify specific treatment effects dependent on strain, we identified differentially expressed genes with tunicamycin or serum+37°C treatment as a function of strain [adjusted *P*-value (*P*_adj_)<0.05]. Of the original 6263 genes, 3429 and 3480 genes exhibited a significant treatment×strain interaction in the tunicamycin and morphogenesis datasets, respectively. Significant genes were clustered to identify groups of genes with unique expression patterns. A group of genes (Fig. 2C; Table S4; cluster 2, 571 genes) was upregulated in tunicamycin-treated wild-type but not in tunicamycin-treated *ire1DX*, an expression pattern expected of UPR target genes. Similarly, a group of genes (Fig. 2D; Table S5; cluster 6, 263 genes) was upregulated in serum+37°C-treated wild-type cells but not in *ire1DX*. These results indicate that these genes capture treatment effects that are dependent on Ire1.

Gene Ontology (GO) analysis revealed that the group of genes induced by tunicamycin in an Ire1-dependent manner were enriched for biological processes known to be crucial in the UPR; this included protein targeting to ER (18 genes; *P*=0.00052), protein localization to Golgi (20 genes; *P*=0.00056) and ER-associated misfolded protein catabolic process (7 genes; *P*=0.00011) (Fig. 2E; Table S6) (Kimata et al., 2006; Travers et al., 2000; Wimalasena et al., 2008). GO analysis for the cluster of genes upregulated in an Ire1- and serum+37°C-dependent manner revealed upregulation of several genes known to form the core filamentation response in *C. albicans* (Fig. 2F; Table S7). This includes *ALS3*, which encodes a cell wall-associated protein (Phan et al., 2007; Zhou et al., 2024), and *DCK1*, which encodes a Rac1 guanine nucleotide exchange factor (Martin et al., 2013). Interestingly, a comparison of the two Ire1-upregulated gene clusters showed that the tunicamycin and serum+37°C treatments each induced a unique transcriptional response. Only 24 genes were identified as being upregulated in both groups of genes (Fig. 2G). Several of these 24 genes have roles in cellular protein localization, protein folding and post-translational modifications, as well as roles in cell wall biogenesis and amino acid metabolism (Table S8). Many of the genes localize to portions of the secretory pathway, including the ER, Golgi and cell membrane, as well as the cell wall. This is logical given the previously highlighted role of Ire1 in cell wall homeostasis (Blankenship et al., 2010).

To further visualize the distinction between the tunicamycin and morphogenesis Ire1-upregulated clusters, differentially expressed genes (*P*_adj_<0.05) were plotted based on their Ire1-dependent fold change in serum and their *P*_adj_ value (Fig. 2H). We observed a clear separation in distribution of tunicamycin cluster 2 genes and morphogenesis cluster 6 genes (blue versus orange dots). This emphasizes the distinct transcriptional programs occurring through Ire1 in response to these two conditions.

To further characterize our findings obtained through RNA sequencing, we performed reverse transcription real-time quantitative PCR (RT-qPCR) on several of the most differentially expressed genes from either dataset. The canonical UPR target genes *KAR2* (Mori et al., 1992) and *PDI1* (Travers et al., 2000) were selected from the tunicamycin-upregulated genes, and *ALS4* and *AHP1* were selected from the serum+37°C-upregulated genes. As expected, the expression of *KAR2* and *PDI1* was increased upon treatment with tunicamycin in wild-type but not in *ire1DX* cells (Fig. S4A,B). Similarly, the expression of morphogenesis genes *AHP1* and *ALS4* increased in wild-type cells treated with serum+37°C but not in *ire1DX* cells (Fig. S4C,D)*.* Neither *KAR2* nor *PDI1* were upregulated in response to treatment with serum+37°C, and *AHP1* was not upregulated in response to tunicamycin, supporting the idea that the Ire1-mediated transcriptional response to both treatments varies greatly.

We also compared the genes upregulated by tunicamycin or serum+37°C in a strain-dependent manner to previously identified UPR and tunicamycin target genes. Microarray analysis was previously used to identify 431 *S. cerevisiae* genes as UPR target genes (Travers et al., 2000). We found that 64 tunicamycin cluster 2 genes and 11 morphogenesis cluster 6 genes had *S. cerevisiae* orthologs in this group of UPR genes (Fig. S4E). Previous transcriptomic analysis of *C. albicans* also identified 2606 genes involved in responding to tunicamycin treatment (Yang et al., 2021); 148 of our tunicamycin cluster 2 genes and 63 of our morphogenesis cluster 6 genes were also found in that dataset (Fig. S4F). In both cases, a greater number of tunicamycin cluster genes than morphogenesis cluster genes matched with genes in the published literature.

An inherent challenge in comparing transcriptomes across strains with different filamentation capacities is that population-level RNA-seq captures the aggregate transcriptional state of morphologically heterogeneous cultures. Because wild-type populations under serum+37°C conditions are predominantly hyphal (∼52%) whereas *ire1DX* populations remain entirely in yeast form (0%), genes identified as Ire1-dependent could in principle reflect morphotype composition rather than direct Ire1 regulation (Fig. 1D,E). Several lines of evidence argue against this interpretation. First, canonical UPR target genes (*KAR2*, *PDI1* and *HAC1*) showed no significant Ire1-dependent fold change under serum+37°C conditions (Fig. 2H), demonstrating that the morphotype difference between strains does not produce a blanket shift in Ire1-associated gene expression. Second, the morphogenesis cluster 6 genes show minimal overlap with established UPR gene sets from both *S. cerevisiae* and *C. albicans* (Fig. S4E,F), indicating that they represent a genuinely distinct transcriptional program rather than an artifact of comparing populations with different stress levels. Although single-cell RNA-seq would in principle provide morphotype-resolved transcriptomes, this approach remains technically challenging for filamentous *C. albicans* populations owing to the difficulty of dissociating and capturing hyphal cells in standard droplet-based platforms. Nevertheless, the convergence of our population-level, gene-level and cross-strain analyses supports the conclusion that the identified transcriptional programs reflect genuine Ire1-dependent regulation.

**Fig. 3.**
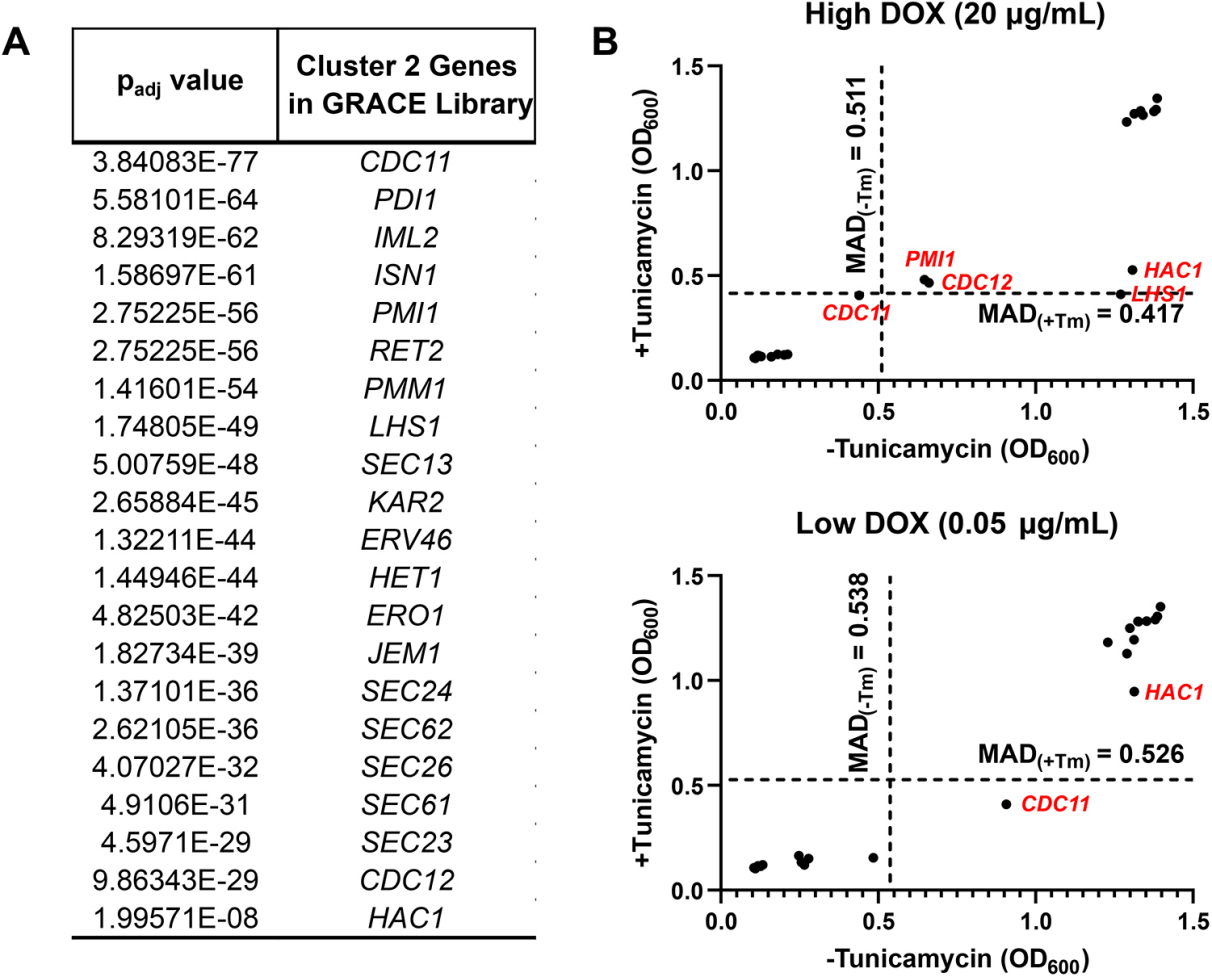
**Screen of top 20 genes upregulated in an Ire1- and tunicamycin-dependent manner identifies genes required for tunicamycin tolerance.** (A) *P*_adj_ values for top 20 differentially expressed genes (plus *HAC1*, which was not in the top 20) in cluster 2 of the tunicamycin RNA sequencing data. Strains containing mutations of each gene were obtained from GRACE or GELC libraries (Fu et al., 2021; Roemer et al., 2003). GRACE parent strain CaSS1 was included as a control. (B) Strains were grown overnight with low concentrations of doxycycline (DOX; 0.05 μg/ml) or high concentrations of DOX (20 μg/ml) at 30°C. Cells were then cultured in YPD containing the same concentration of DOX with or without 1.5 μg/ml tunicamycin and OD_600_ was measured after 24 h. Data points are the average of technical quadruplicates, and dotted lines indicate the median absolute deviation (MAD) cut off values for each condition (Lee et al., 2022). Genes of interest are highlighted in red.

To determine the requirement for the identified Ire1-regulated genes in tunicamycin tolerance, 20 of the genes most differentially expressed with tunicamycin treatment in wild-type but not in *ire1DX* (cluster 2 genes) were selected for further analysis (Fig. 3A). *HAC1* was not one of the top 20 hits, but given its prominent role in the UPR, it was included in the set of assessed genes (Cox and Walter, 1996). Based on previous microarray data (Thibault et al., 2011), 13 of these 21 genes are known UPR target genes in *S. cerevisiae* (Table S9). To obtain *C. albicans* strains with decreased expression of each of these genes, strains were selected from the publicly available *C. albicans* gene replacement and conditional expression (GRACE) library and its associated expansion library (GRACE expanded library collection; GELC). The GRACE method of gene repression allows for the transcriptional repression of tetracycline promoter-regulated genes. In total, the library contains ∼3000 mutants, with the GELC expansion adding 866 more (Fu et al., 2021; Roemer et al., 2003). Our mini-library contained four replicates of each selected strain from the GRACE and GELC libraries. The strains were grown in the presence of low (0.05 μg/ml) or high (20 μg/ml) concentrations of doxycycline to induce repression of both essential and non-essential genes, respectively, followed by a treatment with tunicamycin. Hits were identified by identifying strains with a growth defect when treated with doxycycline and tunicamycin but with no growth defect when treated with doxycycline alone (Fig. 3B) (Lee et al., 2022). This screen identified several genes as being required for tunicamycin tolerance, including *HAC1*, *LHS1*, *PMI1*, *CDC11* and *CDC12. HAC1* is a known UPR target gene required for *S. cerevisiae* to grow in the presence of tunicamycin (Nikawa et al., 1996; Travers et al., 2000). *LHS1* and *PMI1* are also known UPR target genes (Ng et al., 2000; Travers et al., 2000). *LHS1* plays a role in protein translocation into the ER and repair of misfolded proteins (Tyson and Stirling, 2000), and *PMI1* is involved in protein glycosylation (Smith et al., 1995). *CDC11* and *CDC12* encode septins with essential functions in cytokinesis. They have not been previously identified as UPR target genes (Travers et al., 2000); however, ER stress is known to activate a surveillance pathway in *S. cerevisiae* termed the ER stress surveillance (ERSU) pathway. ER stress can activate this pathway, leading to mislocalization of septin subunits including Cdc11 and Cdc12 and resulting in subsequent inhibition of cytokinesis (Babour et al., 2010; Chao et al., 2019).

**Fig. 4.**
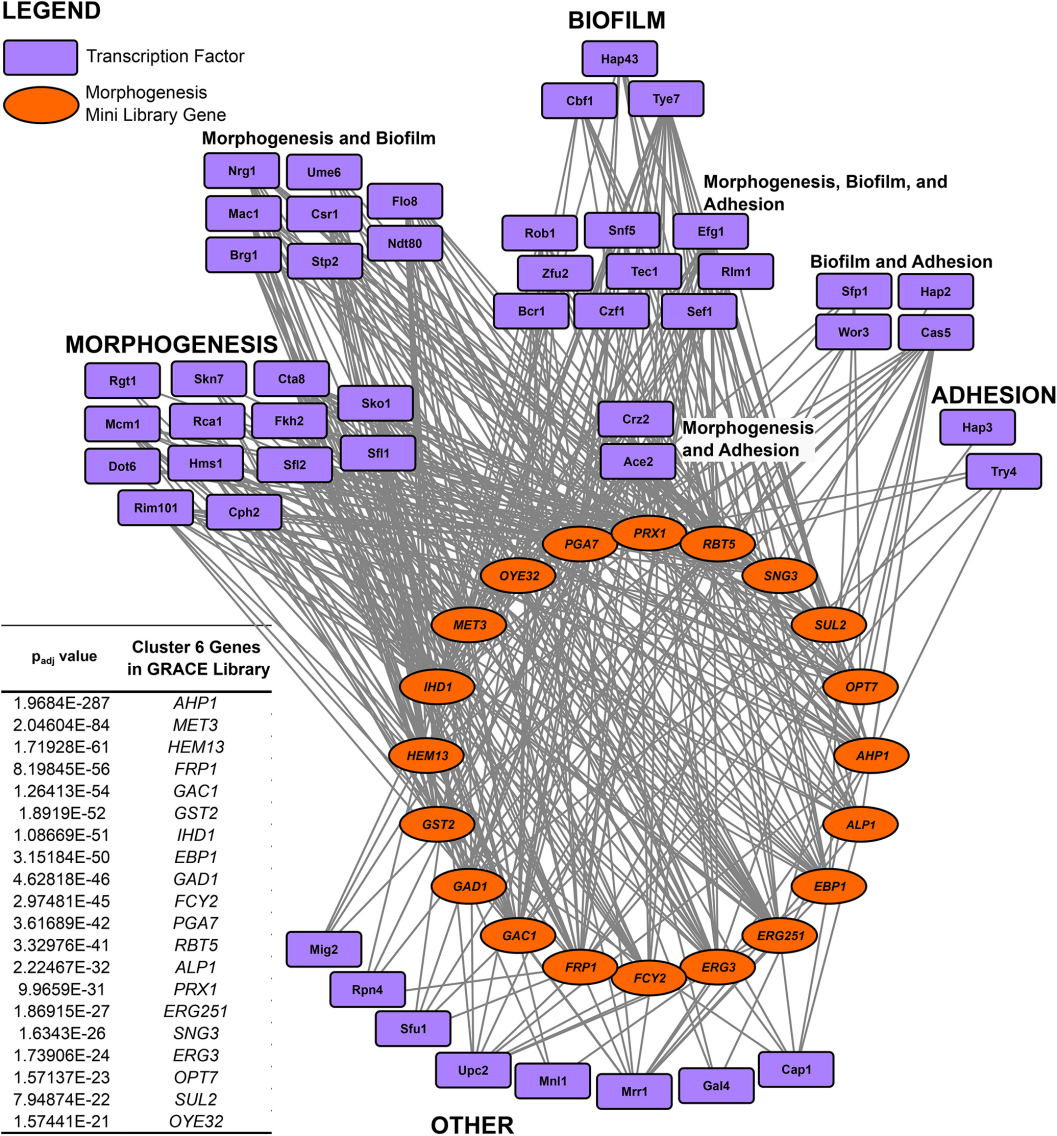
**The top 20 genes upregulated in an Ire1- and serum+37°C-dependent manner, comprising cluster 6, are under control of morphogenesis transcription factors.** The top 20 differentially expressed genes from morphogenesis cluster 6 were subject to transcription factor analysis using PathoYeastrack. 49 transcription factors were identified; an assessment of transcription factor function using the CGD demonstrated that most are involved in regulating morphogenesis, biofilm formation and/or cell adhesion. Purple squares represent transcription factors; orange ellipses represent the top 20 genes. Grey edges represent interactions between transcription factors and genes. Network created in Cytoscape (https://cytoscape.org/). *P*_adj_ values for top 20 differentially expressed genes in cluster 6 of morphogenesis RNA sequencing data shown on bottom left.

We also assessed the top 20 genes upregulated in a serum+37°C- and Ire1-dependent manner (cluster 6 genes; Fig. 4). A preliminary literature review was performed to determine whether these genes have known roles in morphogenesis. All but four genes had reported roles in either filamentation, biofilm formation or both (Table S9) (Alonso-Monge et al., 2010; Braun et al., 2000; Chau et al., 2005; Lee et al., 2014; Miyazaki et al., 2006; Nantel et al., 2002; Nobile et al., 2012; Rosenbach et al., 2010; Seneviratne et al., 2008; Uhl et al., 2003). To further characterize these genes, PathoYeastrack was used to assess common transcription factors controlling their expression (Teixeira et al., 2023). A total of 49 transcription factors were identified as significant (*P*<0.00001; Table S9). The *Candida* genome database (CDG; Lew-Smith et al., 2025) was then used to characterize the regulatory functions of these transcription factors; 32 transcription factors had roles in filamentation, 24 had roles in biofilm formation and 17 had roles in cell adhesion, whereas only 8 had roles unrelated to these functions (Fig. 4). Of note, Hac1 was not one of the top transcription factors identified. This may reflect the genuine absence of Hac1-mediated regulation of these genes, or it might be a limitation of the available databases – no genome-wide Hac1-binding data (e.g. ChIP-seq) currently exists for *C. albicans*, and Hac1 target genes in this organism have been inferred primarily from expression studies rather than direct binding evidence. It is therefore possible that this regulatory interaction has not yet been annotated in the databases referenced for the transcription factor screen. Given that Hac1 was not identified as a key regulator of the morphogenesis gene cluster, we next investigated whether *HAC1* splicing occurs during filamentation and whether Hac1 is required for morphogenesis.

### Limited *HAC1* splicing occurs during morphogenesis

Given the diverging Ire1-regulated transcriptional responses to tunicamycin and filamentation-inducing conditions, we investigated the requirement of *HAC1* for morphogenesis. For this, we developed both *ire1Δ/Δ* and *hac1Δ/Δ* strains using a CRISPR-Cas9 based deletion protocol optimized for *C. albicans* (Halder et al., 2019*)*. DAY286 carrying Cas9 with no guide RNA (DAY286-Cas9) served as the control.

**Fig. 5.**
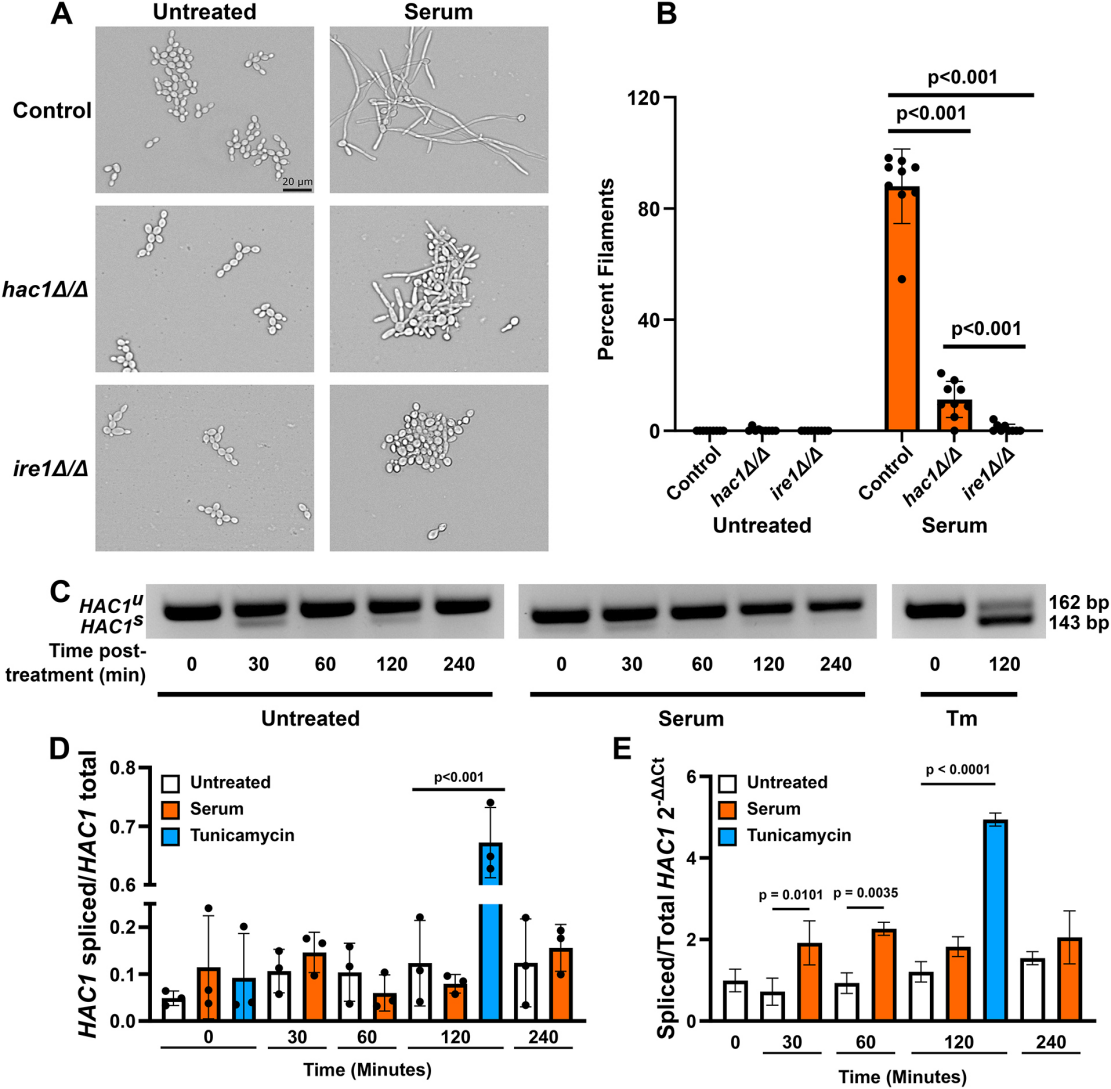
**Limited *HAC1* splicing occurs during morphogenesis.** (A) Control (DAY286-Cas9), *hac1Δ/Δ*, and *ire1Δ/Δ C. albicans* cells were grown in filamentation-inducing 10% fetal bovine serum for 4 h at 37°C and untreated at 30°C as a control. Images obtained using the BioTek Cytation 5 Cell Imaging Multimode Reader. Scale bar: 20 μm, applies to all images. (B) Filamentation was quantified for three biological replicates. Photos were acquired from randomly selected portions of each slide, and round and filamentous cells were counted by hand for each photo. Percentage filaments was calculated and mean±s.d. was plotted. (C) Control (DAY286) cells were grown for 2 h, then treated with 10% serum at 37°C or 1.5 μg/ml tunicamycin at 30°C. Total RNA was isolated from cells at the indicated timepoints after treatment, and RT-PCR was performed using primers flanking the *HAC1* intron, and the RT-PCR products were resolved on a 4% agarose gel. u, unspliced form; s, spliced form. (D) Bands from experiments as in C were quantified using ImageJ (Schneider et al., 2012) to calculate the mean grey value (MGV), and data are presented as a ratio of spliced *HAC1* to total *HAC1* (spliced+unspliced bands). One-way ANOVA with Tukey's multiple comparisons was performed, *n*=3, mean±s.d. plotted. (E) Relative gene expression for spliced *HAC1* and total *HAC1* was assessed in DAY286 *C. albicans* using RT-qPCR. For each strain, a relative expression ratio of spliced to total *HAC1* was calculated using 2^-ΔΔCt^ values. Time represents minutes post treatment. *n*=3, mean±s.d. shown, one-way ANOVA with Tukey's multiple comparisons test was performed.

Next, we performed a morphogenesis assay and found that whereas *ire1Δ/Δ* cells lost the ability to transition to the filamentous form with serum+37°C (0.88% filaments), *hac1Δ/Δ* cells could still form filaments (11.30% filaments) but to a much lesser degree than the control (87.95% filaments; Fig. 5A,B). These *hac1Δ/Δ* filaments appeared much shorter than those of the control, and they did not clump together to form large flocs, as is typical with wild-type cells grown under filamentation-inducing conditions (Bauer and Wendland, 2007) (Fig. S5). These findings were validated using additional *hac1Δ/Δ, ire1Δ/Δ,* and corresponding complemented strains (Fig. S6A,B) (Ramírez-Zavala et al., 2022). To determine whether impaired *HAC1* splicing caused the filamentation defects observed in the *IRE1* deletion strains, an *ire1Δ/Δ* strain carrying constitutively spliced *HAC1* (HAC1*) was grown under filamentation-inducing conditions. HAC1* rescued the filamentation of *ire1Δ/Δ* (Fig. S7A,B). These findings suggest that although *IRE1* is required for morphogenesis, *HAC1* only plays a minor role. This is consistent with previously reported results that show that a *hac1Δ/Δ* strain grown in liquid Spider medium has a filamentation defect that is less pronounced than that in an *ire1Δ/Δ* strain (Lee et al., 2023).

To determine whether *HAC1* splicing occurs in response to filamentation-inducing conditions, cells were treated with serum+37°C, and *HAC1* splicing was assessed over 4 h. As a control, cells treated with tunicamycin showed significant *HAC1* splicing after 120 min. Conversely, cells treated with serum+37°C showed no detectable *HAC1* splicing even after 240 min (Fig. 5C,D). As gel-based methods have the potential to miss minor splicing events, we performed RT-qPCR using two primer sets to detect spliced and total *HAC1*. Again, tunicamycin treatment resulted in significant *HAC1* splicing after 120 min. However, this method also detected minor increases in *HAC1* splicing in serum+37°C-treated cells at 30 and 60 min relative to that in untreated cells; this significant difference disappeared at 120 min onward (Fig. 5E). These findings suggest that whereas *HAC1* is partially involved in regulating morphogenesis, Ire1 is likely regulating morphogenesis through additional mechanisms that are independent of *HAC1* splicing.

### A subset of transcripts encoding secretory proteins is reduced during proteotoxic stress and morphogenesis

We next investigated the mechanism by which Ire1 might be regulating morphogenesis independently of Hac1. In several organisms, Ire1 functions independently of Hac1 through regulated Ire1-dependent decay (RIDD), where ER-localized mRNAs are cleaved by Ire1 and subsequently degraded (Han et al., 2009; Hernández-Elvira et al., 2018; Hollien and Weissman, 2006; Hollien et al., 2009). Although RIDD is a Hac1-independent process mediated by Ire1, it is unclear whether *C. albicans* possesses RIDD. Given that morphogenesis required the Ire1 ribonuclease domain but did not induce *HAC1* splicing, we decided to probe our RNA sequencing data to look for evidence of RIDD.

**Fig. 6.**
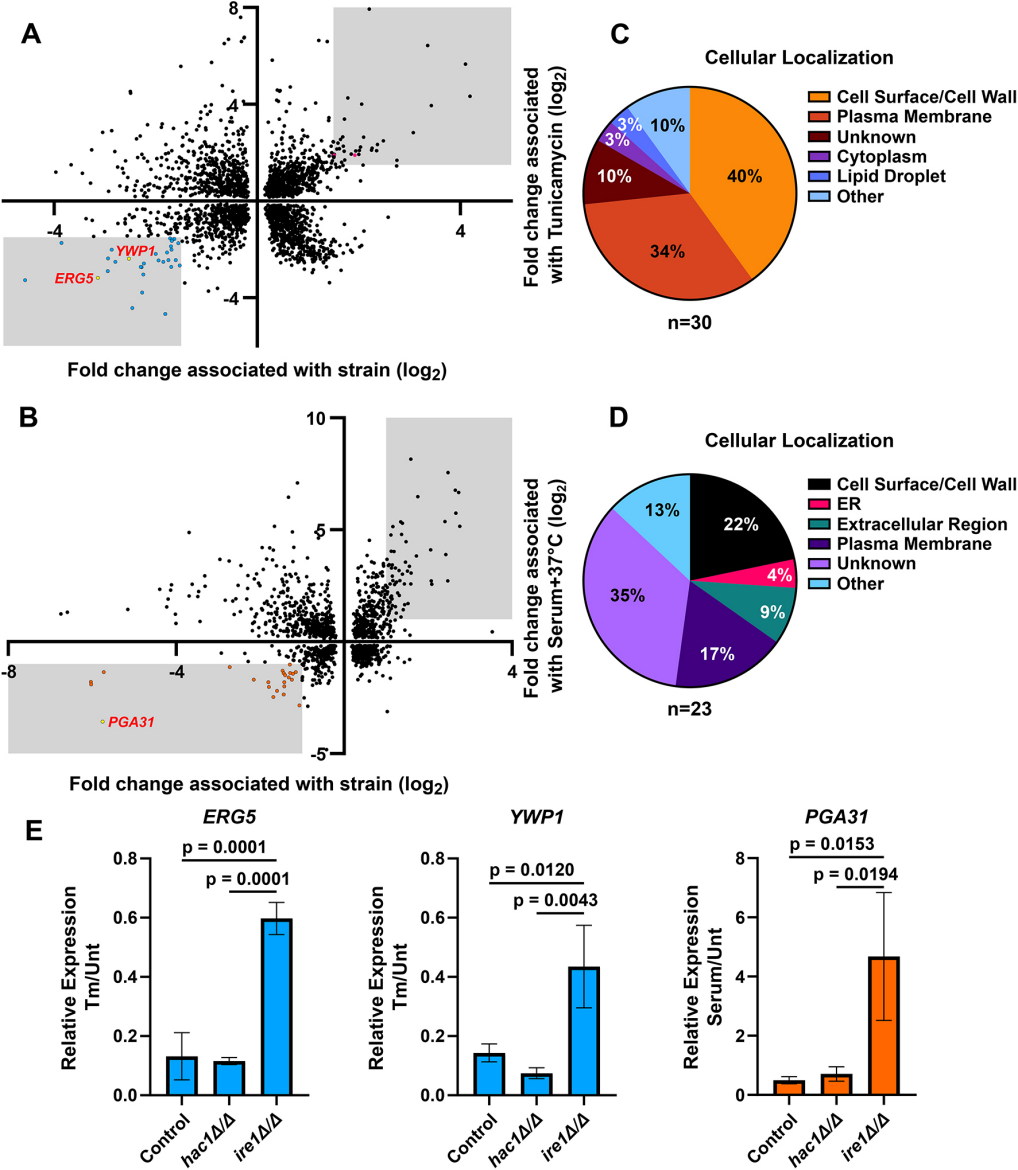
**Genes with decreased expression associated with strain and tunicamycin or serum+37°C treatment encode predominantly secretory proteins.** (A,B) RNA sequencing data was analyzed for genes with decreased expression associated with strain and (A) tunicamycin or (B) serum+37°C treatment. All genes plotted have a significant fold change for both variables (*P*_adj_<0.05). Blue dots represent genes with a decrease in expression greater than 1.5-fold associated with both strain and tunicamycin treatment (*n*=30). Orange dots represent genes with a decrease in expression greater than 1.0-fold associated with both strain and serum+37°C treatment (*n*=23). Pink dots represent *PDI1* and *KAR2*, known UPR target genes. Yellow dots with labels correspond to the genes in E. (C,D) The cellular localization of the protein product for each gene was identified using the CDG for the (C) tunicamycin and (D) morphogenesis datasets. (E) Relative gene expression for *ERG5* and *YWP1* in tunicamycin-treated cells and *PGA31* in serum+37°C-treated cells was assessed using RT-qPCR. Control is DAY286-Cas9. For each strain, a relative expression ratio of treated (tunicamycin or serum+37°C) to untreated cells was calculated using 2^-ΔΔCt^ values. *n*=3, mean±s.d. shown, one-way ANOVA with Tukey's multiple comparisons test performed.

To identify possible RIDD targets, we searched for transcripts that were downregulated upon treatment with tunicamycin or serum+37°C in wild-type cells but not in *ire1DX*, following a similar analysis performed in *S. pombe* (Kimmig et al., 2012). To perform this analysis, we studied the effect of strain in the treated samples (tunicamycin or serum+37°C) and the treatment effect in the wild-type strain. Genes with a significant fold change (*P*_adj_<0.05) for both variables were plotted (Fig. 6A,B). A total of 30 genes had a decrease in expression greater than 1.5-fold associated with both strain and tunicamycin treatment (Fig. 6A, blue dots; Table S10). *PDI1* and *KAR2*, known UPR target genes (Mori et al., 1992; Travers et al., 2000), were identified as having an increase in expression greater than 1.5-fold associated with both strain and treatment (Fig. 6A, pink dots). A total of 23 genes had a decrease in expression greater than 1.0-fold associated with both strain and serum+37°C treatment (Fig. 6B, orange dots; Table S11).

RIDD targets are primarily secretory proteins produced in the ER (Hollien and Weissman, 2006; Hollien et al., 2009; Kimmig et al., 2012). To characterize the transcripts with decreased expression associated with strain and tunicamycin treatment, the CDG was used to identify the protein product and cell component of each gene (Lew-Smith et al., 2025). Analysis of the cellular compartment for the protein product encoded by each gene revealed an enrichment in secretory proteins; 74% of the proteins localize to the cell membrane, cell wall or cell surface (Fig. 6C). Some of these proteins include GPI-linked cell wall proteins, membrane transport proteins, and several membrane-localized ferroxidases. Additionally, 36.7% of these genes were predicted to have a transmembrane domain based on sequence homology. Several genes encoding non-secretory pathway proteins were identified; these genes encode proteins localized to the nucleus, cytoplasm and lipid droplets, and one gene that encodes for a portion of the snoRNP complex (Table S10). A similar analysis was performed for genes with decreased expression associated with strain and serum+37°C treatment, and a similar trend was found; 52% of those genes encode for proteins that localize to the cell surface, cell membrane, ER or extracellular region, and 26% were predicted to have a transmembrane domain (Fig. 6D; Table S11).

To validate potential Ire1-mediated degradation, we assessed transcript levels of candidate genes in wild-type, *hac1Δ/Δ*, and *ire1Δ/Δ* strains under both tunicamycin and filamentation-inducing conditions (Fig. 6E). In wild-type cells treated with tunicamycin, *ERG5* and *YWP1* transcripts showed an expected decrease in the treated versus control ratio, while *PGA31* decreased under filamentation conditions, consistent with Ire1-mediated mRNA degradation. Importantly, these decreases were absent in *ire1Δ*/*Δ* cells for both treatments, confirming that the transcript reduction is dependent on Ire1 function, whereas *hac1Δ*/*Δ* cells showed similar reductions to wild type.

To search for possible Ire1-recognition motifs amongst the identified secretory transcripts, we performed a motif search using STREME, a MEME suite tool that can detect ungapped motifs enriched in a set of sequences (Bailey, 2021, 2015). STREME analysis of the 30 tunicamycin candidate coding sequences identified several enriched motifs relative to all *C. albicans* coding sequences (Table S12). One of the most significant motifs (ACTGCTGCT; *P*=4.00×10^6^) was present in the majority of candidate genes and contained a core GCTG element. However, comparison with known motif databases using Tomtom (Gupta et al., 2007) revealed that this element corresponds to recognition sites for well-characterized transcription factors, including the yeast cell-separation regulators *SWI5* and *ACE2* (Simon et al., 2001) in fungal databases (Table S13). Similarly, STREME identified several other motifs in the serum+37°C gene set, including the ACTGCTGCT motif found in the tunicamycin dataset, but Tomtom analysis again matched these to known transcription factor recognition sites. Based on these analyses, we were unable to identify a clear candidate for a unique Ire1 cleavage motif in either set of transcripts. This is consistent with findings in *C. glabrata*, where RIDD activity has been confirmed but no consensus Ire1 recognition sequence has been identified (Miyazaki et al., 2013).

To determine whether the decreased transcript levels for our candidate genes were caused by increased degradation or decreased transcription, we utilized thiolutin as a transcriptional inhibitor and monitored gene levels over a period of 2 h (Kebaara et al., 2006). However, thiolutin treatment alone caused substantial decay of *ERG5* transcripts and appeared to induce expression of the UPR target gene *INO1*, suggesting that thiolutin itself might activate stress responses that confound the interpretation of Ire1-dependent decay (Fig. S8). This is consistent with recent findings that thiolutin has complex pleiotropic effects *in vivo*, including induction of oxidative stress, Tor pathway inhibition and disruption of metal homeostasis, beyond its role as a transcriptional inhibitor (Qiu et al., 2024). Although *ERG5* decay was modestly more pronounced in strains with functional Ire1 compared to *ire1Δ*/*Δ* under tunicamycin+thiolutin conditions, these differences were not sufficient to conclusively demonstrate Ire1-mediated mRNA degradation (Fig. S8). Future studies using alternative approaches, such as *in vitro* cleavage assays with a purified Ire1 RNase domain, or methods to map specific endonucleolytic cleavage sites, will be necessary to definitively determine whether Ire1 directly cleaves these transcripts.

### Ire1 regulates the cell wall stress response through both Hac1-dependent and Hac1-independent mechanisms

We next explored another mechanism by which Ire1 could be regulating morphogenesis independently of Hac1. We assessed genes upregulated in an Ire1- and serum+37°C-dependent manner (cluster 6 genes) using Gene Ontology analysis for cellular compartment and identified an enrichment in genes encoding cell wall proteins (Table S7). Cell wall protein production is known to increase during morphogenesis to support growth at the hyphal tip (Mundodi et al., 2021; Weiner et al., 2019). Cell wall proteins are also linked to Ire1 and the UPR through the cell wall integrity pathway (CWI). The CWI is a stress pathway responsible for resolving cell wall damage (Sanz et al., 2017). During ER stress, the transport of proteins to the cell wall can become impaired and cause cell wall perturbations, leading to cell wall stress. Alternatively, cell wall stress can cause the cell to increase its protein folding capacity in an attempt to alleviate cell wall damage, leading to increased ER stress (Krysan, 2009). Given the known importance of Ire1 in both morphogenesis and cell wall integrity (Blankenship et al., 2010; Scrimale et al., 2009; Sircaik et al., 2021), we investigated whether morphogenesis could be rescued in *ire1Δ/Δ* cells by treatment with the osmotic stabilizer sorbitol. Sorbitol has been shown to rescue cell wall defects in various yeast (Chen et al., 2005; Cheon et al., 2011; Sircaik et al., 2021). We did not observe a significant increase in the percentage of filaments when *ire1Δ/Δ* was grown with sorbitol (Fig. S9).

**Fig. 7.**
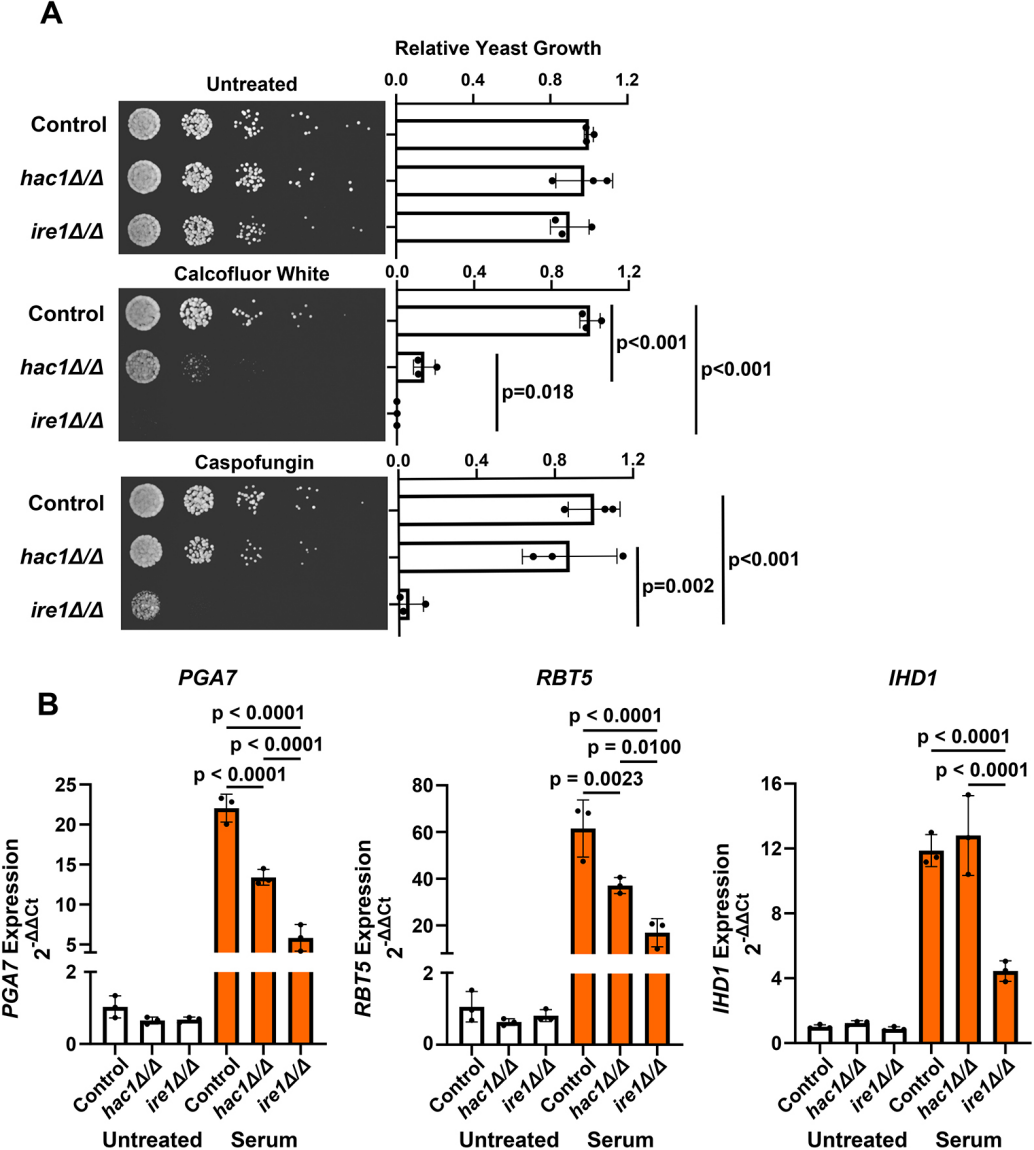
***HAC1* is not required for cell wall stress to the same degree as *IRE1*.** (A) Control (DAY286-Cas9), *hac1Δ/Δ*, and *ire1Δ/Δ C. albicans* cells were spotted on plates containing 10 μg/ml Calcofluor White and 0.01 μg/ml caspofungin. Images were acquired after 24 h. Statistical analysis performed using a one-way ANOVA with Tukey's multiple comparisons test, *n*=3, mean±s.d. plotted. (B) Relative expression of GPI-anchored cell wall genes. One-way ANOVA with Tukey's multiple comparisons test. Mean±s.d. shown, *n*=3.

Next, we sought to determine whether Hac1 is required for the Ire1-dependent regulation of cell wall integrity. When grown on the cell wall stressors Calcofluor White and caspofungin, *hac1Δ/Δ* cells grew less than the Cas9-control but more than *ire1Δ/Δ* cells (Fig. 7A). These findings were validated using additional *hac1Δ/Δ* and *ire1Δ/Δ* mutants and their respective complements (Fig. S10A). As expected, these deletion strains also show a high sensitivity to tunicamycin, which is rescued by complementation (Fig. S10A). To further clarify whether a lack of *HAC1* splicing caused the observed sensitivity of *IRE1* deletion strains to cell wall stressors, we grew strains carrying constitutively spliced *HAC1* (HAC1*) in the presence of Calcofluor White and caspofungin. HAC1* rescued the growth of *ire1Δ/Δ* in the presence of both agents, indicating that cell wall stress tolerance – unlike the upregulation of cell wall genes described below – is at least partially Hac1-dependent (Fig. S11A). We note, however, that both ire1Δ/Δ+HAC1* and ire1Δ/Δ+IRE1-WT+HAC1* cells exhibited substantially elevated total *HAC1* levels (Fig. S11B), so we cannot exclude the possibility that this rescue reflects increased Hac1 dosage in addition to constitutive splicing (Miyazaki et al., 2013; Ramírez-Zavala et al., 2022). This elevated *HAC1* expression is consistent with the removal of regulatory elements in the *HAC1* intron that normally repress translation and promote mRNA degradation of the unspliced transcript, resulting in stabilization and accumulation of the constitutively spliced form (Cox and Walter, 1996; Matabishi-Bibi et al., 2022).

We also assessed the expression of cell wall genes in *hac1Δ/Δ* and *ire1Δ/Δ* cells under filamentation conditions. *PGA7*, *RBT5* and *IHD1* were selected from the group of genes upregulated in an Ire1- and serum+37°C-dependent manner. All three encode glycophosphatidylinositol (GPI)-linked cell wall proteins and are known to be upregulated in response to filamentation cues (Azadmanesh et al., 2017). We identified a similar trend; *IRE1* was required for the expression of all three genes under morphogenesis-inducing conditions, whereas *HAC1* was either partially required (*PGA7*, *RBT5*) or not required at all (*IHD1*; Fig. 7B and Fig. S10B). Thus, the transcriptional upregulation of GPI-anchored cell wall genes during morphogenesis is independent of Hac1, in contrast to cell wall stress tolerance, which retains a partial Hac1 requirement (Fig. S11A). These two outputs are therefore mechanistically separable. These results further support a model in which Ire1 regulates cell wall remodeling during morphogenesis through mechanisms that extend beyond its canonical role in activating the Hac1 transcriptional program.

## DISCUSSION

### Hac1-independent regulation of hyphal growth by Ire1

Although our results confirm the canonical role of Ire1 in activating the UPR through Hac1 during ER stress, the stark disconnect between the essential role of Ire1 in morphogenesis and the partial requirement for Hac1 suggests that alternative Ire1-dependent pathways drive filamentous growth in *C. albicans*. Previous studies have established separable functions for Ire1 and Hac1, including roles in iron uptake (Ramírez-Zavala et al., 2022), manganese tolerance (Henry et al., 2024) and colony morphology (Lee et al., 2023). Our study extends these observations in three ways: first, genome-wide transcriptomic analysis reveals that the Ire1-dependent programs during proteotoxic stress and morphogenesis are fundamentally distinct, with only 24 of over 800 Ire1-upregulated genes shared between conditions; second, we provide the first systematic identification of Ire1-dependent and Hac1-independent reduction of transcripts encoding secretory pathway proteins in *C. albicans*, consistent with RIDD activity in other fungi; and third, we establish cell wall integrity as a specific Hac1-independent output of Ire1, with Ire1, but not Hac1, required for upregulation of GPI-anchored cell wall proteins during filamentation. The UPR displays remarkable plasticity, with target gene expression tailored to the type of stress (Thibault et al., 2011), which would explain why proteotoxic stress and morphogenesis elicit distinct responses despite both requiring Ire1.

We note that our comparison employed different temperatures (30°C versus 37°C) and treatment durations (2 h versus 4 h), raising the possibility that some transcriptional divergence reflects these variables. However, RT-qPCR confirmed that canonical UPR targets (*KAR2* and *PDI1*) were not induced by serum+37°C and the morphogenesis gene *AHP1* was not induced by tunicamycin, demonstrating condition-specific regulation. Furthermore, morphogenesis cluster 6 genes showed minimal overlap with established tunicamycin-induced gene sets from both *S. cerevisiae* and *C. albicans* (Fig. S4E,F).

Our findings are in accordance with previously reported requirements for Ire1 in morphogenesis (Blankenship et al., 2010; Sircaik et al., 2021; Zhao et al., 2025). Deletion of *HAC1* decreased filamentous growth in liquid Spider medium and delayed wrinkly colony formation on solid medium, but in both cases less severely than *IRE1* deletion (Lee et al., 2023). Other Hac1-independent roles include Ftr1 plasma membrane localization (Ramírez-Zavala et al., 2022) and manganese tolerance (Henry et al., 2024). In other fungi, Ire1 functions independently of Hac1 through RIDD (Hernández-Elvira et al., 2018). *C. glabrata* Ire1 possesses kinase and ribonuclease activity but does not facilitate *HAC1* splicing; its transcriptional response to ER stress instead relies on Ca^2+^ signaling and the Slt2 MAPK pathway (Hernández-Elvira et al., 2018; Miyazaki et al., 2013), with Ire1 mediating RIDD independently of *HAC1* (Miyazaki et al., 2013). Our finding that morphogenesis requires the Ire1 ribonuclease domain but is associated with only limited *HAC1* splicing led us to question whether *C. albicans* possesses RIDD (Figs 1F and 5C–E).

In other fungi, such as *S. pombe*, which lacks *HAC1* and relies solely on RIDD, transcripts targeted for decay primarily encode ER-directed proteins (Hernández-Elvira et al., 2018; Hollien and Weissman, 2006; Kimmig et al., 2012). Similarly, *C. glabrata* RIDD targets predominantly encode GPI-anchored cell wall and membrane proteins (Miyazaki et al., 2013). Consistent with this, over 70% of the genes we identified with decreased expression associated with strain and tunicamycin treatment encode proteins localizing to the cell surface, cell wall and plasma membrane (Fig. 6A,C), with a similar pattern for serum+37°C treatment (Fig. 6B,D). We attempted to distinguish between reduced synthesis and increased decay using thiolutin as a transcriptional inhibitor (Kebaara et al., 2006), but thiolutin alone caused substantial *ERG5* decay and induced expression of the UPR target *INO1* (Fig. S8), consistent with its known pleiotropic effects (Qiu et al., 2024). Although a modest Ire1-dependent difference in *ERG5* decay was observed under tunicamycin+thiolutin conditions, this was insufficient to conclusively demonstrate endonucleolytic cleavage. Future experiments such as *in vitro* cleavage assays with purified Ire1 RNase domain or approaches to map cleavage sites (Kimmig et al., 2012) will be needed to resolve whether Ire1 directly cleaves these transcripts. We also searched for shared motifs representing an Ire1-recognition site but were unable to identify a clear candidate. This is consistent with what occurs in *C. glabrata*, where RIDD targets lack a consensus site (Miyazaki et al., 2013; Walter and Ron, 2011), although organisms such as mammals and *S. pombe* do possess defined Ire1 cleavage motifs (Bashir et al., 2021; Kimmig et al., 2012; Moore and Hollien, 2015; Oikawa et al., 2010).

Although we cannot definitively confirm RIDD in *C. albicans*, the Ire1-dependent decrease in transcripts encoding secretory proteins during both proteotoxic stress and morphogenesis likely reduces the translational burden on the ER (Bashir et al., 2021; Coelho and Domingos, 2014). For tunicamycin, this would aid recovery from protein misfolding caused by disrupted N-linked glycosylation (Barnes et al., 1984; Thomas et al., 2015). During morphogenesis, the minor *HAC1* splicing at early timepoints (Fig. 5E) suggests a low level of ER stress that could benefit from reduced transcript load. Additionally, many filamentation genes show reduced translational efficiency despite transcriptional induction during morphogenesis (Mundodi et al., 2021), suggesting widespread translational fine-tuning. The Ire1-dependent decrease in secretory transcripts could thus facilitate morphogenesis by redirecting cellular resources toward energy-intensive processes, such as rapid cell wall expansion and polarized development, while providing post-transcriptional control to complement the transcriptional programs driving the yeast-to-hyphal transition.

### Cell wall integrity and morphogenesis

**Fig. 8.**
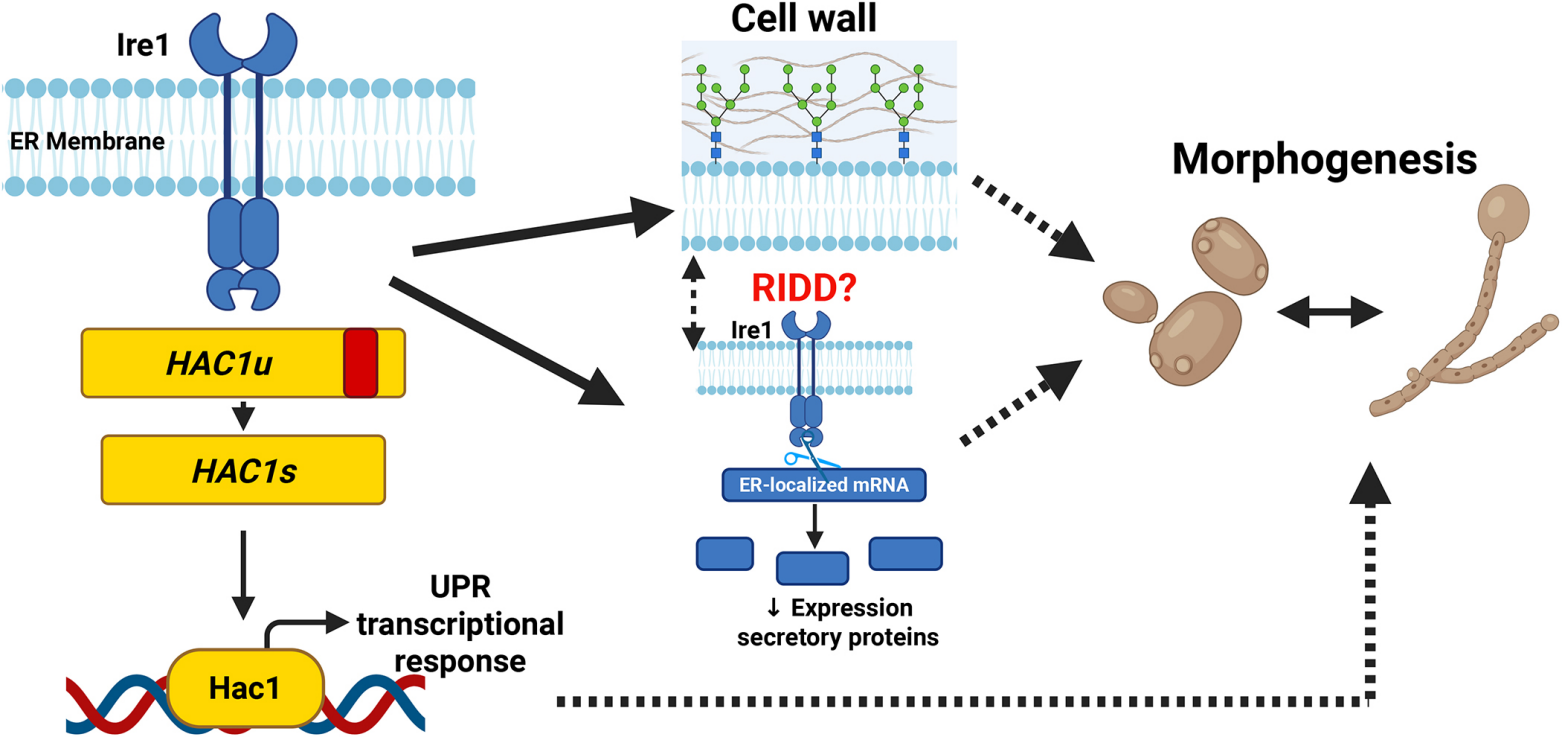
**Diverse functions of Ire1 drive morphogenesis in *C. albicans*.** Proposed model depicting the multiple mechanisms by which Ire1 regulates morphogenesis in *C. albicans*. Unlike the canonical ER stress response, filamentation-inducing conditions trigger only limited *HAC1* splicing, yet fully require Ire1 for hyphal development. Ire1 regulates morphogenesis through three potential mechanisms: (1) Hac1-independent regulation of cell wall integrity genes, including upregulation of cell wall proteins and cell wall stress tolerance; (2) Ire1-dependent decreases in mRNAs encoding secretory pathway proteins – particularly those localizing to the cell membrane and cell wall; and (3) a Hac1-dependent response that is only partially required for filamentation. The kinase and nuclease domains of Ire1 are both essential for morphogenesis. This model highlights the mechanistic divergence between the Ire1-dependent transcriptional programs activated during proteotoxic stress versus those required for morphogenesis, with minimal overlap between the two responses. Created in BioRender by Stack-Couture, S., 2026. https://BioRender.com/cv7skbd. This figure was sublicensed under CC-BY 4.0 terms.

We also explored cell wall stress as a connection between Ire1 and morphogenesis, given that Ire1 is part of the circuitry controlling cell wall homeostasis in *C. albicans* (Blankenship et al., 2010). Our RNA sequencing revealed cell wall genes upregulated during morphogenesis, and RT-qPCR confirmed a requirement for *IRE1* but not *HAC1* in their upregulation. *HAC1* was required for growth in the presence of cell wall stressors to a lesser degree than *IRE1* (Fig. 7A), and constitutively spliced HAC1* partially rescued *ire1Δ/Δ* on these agents (Fig. S11A), indicating that cell wall stress tolerance retains a partial Hac1 requirement. In contrast, the upregulation of GPI-anchored cell wall genes during morphogenesis was Hac1 independent (Fig. 7B). The distinction between these outputs is consistent with a previous finding showing that an *IRE1*-depleted strain was sensitive to Calcofluor White without undergoing *HAC1* splicing (Sircaik et al., 2021) and indicates that the contribution of Ire1 to the cell wall stress response cannot be attributed to a single Hac1-dependent or -independent mechanism. Because the ER is the primary site of cell wall protein production, impaired Ire1 function disrupts cell wall protein synthesis and integrity (Blankenship et al., 2010; Krysan, 2009; Scrimale et al., 2009), which might explain the impaired filamentation in *IRE1*-deficient strains, as the yeast-to-hyphal transition requires rapid cell wall expansion at the hyphal tip (Weiner et al., 2019). However, the less severe filamentation defect for *hac1Δ/Δ* compared to *ire1Δ/Δ* cells suggests that canonical UPR target gene upregulation is not the primary mechanism, and that Ire1 likely engages additional downstream targets to facilitate cell wall stress adaptation. An important question is whether the cell wall integrity and secretory transcript degradation phenotypes represent independent Ire1 outputs or are mechanistically linked. Many Ire1-dependent downregulated transcripts encode cell surface and cell wall proteins (Fig. 6C,D), suggesting that selective mRNA degradation could directly remodel the cell wall proteome during the yeast-to-hyphal transition. Conversely, the Ire1-dependent upregulation of cell wall biosynthesis genes (Fig. 7B) suggests a parallel transcriptional program operating alongside any post-transcriptional regulation. Distinguishing between these possibilities will require targeted disruption of individual candidate genes to determine whether their degradation is functionally required for cell wall remodeling during morphogenesis. These findings align with work showing that Ire1 maintains basal ER functionality independent of Hac1 – Ire1 is essential for proper plasma membrane localization of the iron permease Ftr1, with *ire1Δ* mutants exhibiting Ftr1 retention in the ER despite the absence of *HAC1* splicing (Ramírez-Zavala et al., 2022). This quality control function might be particularly important during morphogenesis, when rapid cell wall expansion requires efficient trafficking of secretory pathway proteins. The inability of *ire1Δ/Δ* but not *hac1Δ/Δ* mutants to upregulate cell wall biosynthesis genes during filamentation suggests that the role of Ire1 in maintaining secretory pathway homeostasis is essential for supporting the protein flux required for hyphal development, even in the absence of overt ER stress (Fig. 8).

### Differential Ire1 signaling and perspectives

A central question raised by our findings is how Ire1 generates fundamentally different outputs depending on the activating stimulus. Recent work in mammalian systems provides a mechanistic framework: *HAC1* (*XBP1* in mammals) cleavage requires cooperative oligomerization of multiple IRE1 subunits, whereas RIDD can be performed by individual dimers without cooperativity (Tam et al., 2014), and the two RNase outputs are temporally separable (Bright et al., 2015). Moreover, mammalian IRE1α possesses a non-canonical RIDD mode (RIDDLE) that cleaves mRNAs without a consensus endomotif (Le Thomas et al., 2021). The relationship between *HAC1* or *XBP1* splicing and RIDD varies across evolution – from *S. cerevisiae* (splicing only) to *S. pombe* (RIDD only) (Hollien and Weissman, 2006; Kimmig et al., 2012; Le Thomas et al., 2021). Our data suggest that *C. albicans* Ire1 retains functional *HAC1* splicing – as demonstrated by robust splicing during tunicamycin treatment and cross-species complementation of *S. cerevisiae* (Fig. S3) – while also exhibiting Ire1-dependent and Hac1-independent decreases in secretory transcripts reminiscent of RIDD. The minor and transient *HAC1* splicing detected early during serum+37°C treatment (Fig. 5E) is consistent with a threshold model in which morphogenesis-associated signals activate Ire1 to a level that permits limited *HAC1* splicing and alternative outputs, but falls short of the sustained activation triggered by proteotoxic stress. Condition-specific cofactors might also direct Ire1 toward different substrates; Ire1 activates the HOG-MAPK pathway during ER stress in *C. albicans* (Husain et al., 2021), and it remains possible that Ire1 responds to secondary perturbations in ER homeostasis arising from the increased secretory demand during rapid hyphal extension.

Several important limitations and future directions merit discussion. First, no genome-wide Hac1 occupancy data (e.g. ChIP-seq) exists for *C. albicans*; such data would clarify whether the morphogenesis genes we identified as apparently Hac1 independent are truly outside the Hac1 regulon (Travers et al., 2000). Second, we did not examine whether our kinase-dead mutant maintained endoribonuclease activity that was equivalent to wild type. Although Ire1 autophosphorylation has been shown to be dispensable for endoribonuclease activation but required for deactivation (Chawla et al., 2011; Han et al., 2009; Rubio et al., 2011), further studies are needed to definitively separate these two functions in the context of morphogenesis. Third, a major constraint is the lack of direct evidence that Ire1 endonucleolytically cleaves the secretory transcripts we identify as potential targets. Future studies using *in vitro* cleavage assays with purified Ire1 (Lee et al., 2011), or northern blot analysis in Ski complex deletion mutants to detect cleavage intermediates (Brown et al., 2000; Kimmig et al., 2012), will be necessary to resolve this question.

It would also be of interest to explore whether the Hac1-independent role of Ire1 in morphogenesis persists under other filamentation-inducing conditions. Transcriptional responses to different inducing stimuli vary considerably (Azadmanesh et al., 2017), yet *IRE1* was identified as a core gene required for filamentation across multiple conditions in that study, suggesting a broadly conserved role. Different stimuli likely engage the UPR to varying degrees; for instance, proteasome inhibition induces morphogenesis through accumulation of misfolded proteins (Hossain et al., 2020), which would be expected to robustly activate *HAC1* splicing, suggesting that Hac1-dependent and Hac1-independent mechanisms might operate in parallel, with their relative contributions varying by stimulus. Requirements for specific filamentation genes can also differ between *in vivo* and *in vitro* conditions (Wakade et al., 2021, 2022, 2024), underscoring the need to test these pathways under physiologically relevant conditions.

Given that Ire1 is essential for *C. albicans* morphogenesis and pathogenesis, and considering the extensive development of IRE1-targeting small molecules for mammalian systems (Grandjean and Wiseman, 2020), Ire1 emerges as a promising druggable target for antifungal therapy, with opportunities to develop fungal-specific inhibitors that could serve as combination therapies with current antifungal agents (Kamath et al., 2024; Wang et al., 2025).

## MATERIALS AND METHODS

### Yeast strains and cell cultures

All *C. albicans* strains used in this study are listed in Table S1. All strains were grown from frozen glycerol stocks on YPD (1% yeast extract, 2% peptone, 2% dextrose; Thermo Fisher Scientific) or selective synthetic complete (SC; with appropriate amino acids, Thermo Fisher Scientific) medium plates for 48 h at 30°C. All liquid cultures were grown in triplicate overnight in 5 ml of YPD or SC medium at 30°C in a rotating drum.

### Reagents

Stock solutions of tunicamycin (5 mg/ml in DMSO; Millipore Sigma, Burlington, MA, USA), fetal bovine serum (100%; Millipore Sigma), caspofungin (1 mg/ml in H_2_O; Millipore Sigma), dithiothreitol (DTT; Promega, Madison, WI, USA), thiolutin (10 mg/ml in DMSO; Millipore Sigma) and Calcofluor White (10 mg/ml in H_2_O; fluorescence brightener 28; Millipore Sigma) were prepared and diluted to concentrations indicated in the text.

### Strains and plasmids design

All primers and plasmids used in this study are listed in Table S2. All plasmids generated in this paper underwent whole plasmid sequencing by Plasmidsaurus using Oxford Nanopore Technology with custom analysis and annotation.

*ire1DX* was transformed with the indicated plasmids to produce *ire1DX+IRE1-WT* and other *ire1DX* variants following the protocol described in Sircaik et al. (2021). Briefly, plasmids were cut with NruI to direct insertion into the *C. albicans HIS1* locus. Correct insertion into the locus was confirmed using *IRE1* Comp primers (Table S2) (Sircaik et al., 2021).

CRISPR strains were designed following the protocol in Halder et al. (2019). Briefly, EuPaGDT (http://grna.ctegd.uga.edu/; Tarleton Research Group, The Center for Tropical and Emerging Global Diseases, The University of Georgia, USA) was used to design small guide RNAs (sgRNA) specific to the gene of interest. The sgRNA sequences and gene-specific homology arms were incorporated into a Gene Drive fragment synthesized by IDT (Integrated DNA Technologies, Inc.; Table S3). The gene drive was cloned into the *pRS252-Neut5L-CaCas9* backbone using Gibson Assembly, and the resulting plasmid was amplified in *Escherichia coli*. Purified plasmids were sequenced with Plasmidsaurus to confirm correct integration of the gene drive. Plasmids were cut with PacI and transformed into the indicated *C. albicans* strain. Transformation success was assessed using gene drive deletion primers to confirm deletion of the gene of interest (Table S2).

The ire1Δ/Δ+HAC1* and ire1Δ/Δ+IRE1-WT+HAC1* strains were constructed by excising the insert contained in *pHAC1ex1E1* with ApaI and SacII (Ramírez-Zavala et al., 2022). The resulting fragment was transformed into *ire1Δ/Δ* and *ire1Δ/Δ+IRE1-WT* to replace an *ADH1* allele in either strain.

*pRS313 ScPromoter-CaIRE1* was constructed by amplifying *C. albicans IRE1* (*CaIRE1*) from SC5314 genomic DNA using *CaIRE1* EcoRI primers (Table S2). The resulting fragment was cut with EcoRI and cloned into a vector containing the *S. cerevisiae IRE1* promoter but no open reading frame (*pRS313-ScIRE1Promoter*; Table S3) to generate *C. albicans IRE1* under control of the *S. cerevisiae IRE1* promoter. *pRS313-ScIRE1Promoter* was generated by cloning the *S. cerevisiae IRE1* promoter (synthesized by Genewiz, Azenta Life Sciences, South Plainfield, NJ, USA; Table S3) cut with EcoRI/XhoI into *pRS313*.

### Spot assays

*C. albicans* cells from overnight cultures were diluted to an optical density at 600 nm (OD_600_) of 0.1 and then serially diluted 1:5 in a 96-well plate using appropriate medium. Four sequential 1:5 serial dilutions were prepared in the 96-well plate, and a 48-pin replica plater was used to spot the cells onto agar plates containing the indicated concentrations of drugs. Plates were incubated overnight at 30°C and imaged using a SP Imager (S&P Robotics, North York, ON, Canada).

Quantification was performed either using an accompanying liquid growth assay (Fig. 1) or with ImageJ (Schneider et al., 2012), following the protocol described in Petropavlovskiy et al. (2020). Briefly, images were converted to 8-bit format and background noise was subtracted using the rolling ball algorithm. A mean grey value was calculated for the background, and grey values of cell spots were measured using the same dilution for each strain. The mean grey value of the background was subtracted from the grey value of each spot, and the resulting values were used for quantification (Petropavlovskiy et al., 2020). Specific normalization and statistical analysis information is described in the corresponding figure legends. Quantification was performed using IBM SPSS Statistics version 30.0.0.0 (172) for Windows (IBM Corporation, Armonk, NY, USA).

### Liquid growth assays

Overnight cultures of *C. albicans* grown in triplicate were diluted to an OD_600_ of 0.1 and then treated with the indicated drugs. 200 μl aliquots of cells were distributed to a 96-well plate. The plates were placed in an Epoch 2 Microplate Reader (BioTek, Winooski, VT, USA) and OD_600_ readings were measured every 15 min for 24 h. Growth curves were generated and area under the curve was calculated using GraphPad Prism version 10.5.0 for Windows (GraphPad Software, Boston, MA, USA). Statistical analysis was performed using a one-way ANOVA followed by Tukey's multiple comparisons test in IBM SPSS Statistics.

### Filamentation assays and quantification

Cells were grown overnight in triplicate in YPD or SC medium. In the morning, cells were diluted to OD_600_ of 0.2 in fresh medium and left untreated or treated with 10% fetal bovine serum. Samples were then split into two tubes for growth at both 30°C and 37°C. All samples were incubated for 4 h. Samples were plated on microscope slides, and imaging was performed using a BioTek Cytation 5 Cell Imaging Multimode Reader.

A protocol for quantifying percent filamentation was developed using a combination of two published protocols (Bettauer et al., 2022; Wakade et al., 2021). For sample preparation, samples were diluted so that, at a medium magnification, there were no more than ∼100 cells in the field of view. Image acquisition was undertaken using the BioTek Cytation 5 Cell Imaging Multimode Reader microscope; three images were taken per replicate for a total of nine photos per treatment condition. Images were taken at random locations throughout the slide, and images with fewer than 10 cells were rejected. The field of view was adjusted as needed to ensure cells and filaments were fully visible and not cut off on the edge. For each photo, cells were classified as either round or filamentous. Round cells were defined as being a single round cell, or a round cell with an extension less than twice the diameter of the mother cell body (Fig. S1A). Buds are not considered unique cells and are counted as part of the mother cell. Filamentous cells were defined as a mother cell with a filamentous projection at least twice the length of the mother cell body (Fig. S1B). Total round and total filamentous cells were counted by an observer aware of the experimental setup. For large flocs of cells, the center clump of the floc was excluded from the quantification given that the cells become too dense to accurately classify cell type. The percentage of filamentous cells was calculated for each photo and average percentage of filamentous cells for each sample was plotted with standard deviation. Statistical analysis was performed using a one-way ANOVA followed by Tukey's multiple comparisons test in IBM SPSS Statistics.

### RNA purification and quality control

DAY286 and *ire1DX* were cultured overnight in quadruplicate. In the morning, cells were diluted to an OD_600_ of 0.2 and grown for 90 min to obtain log phase cells. Cells were then treated with 1.5 μg/ml tunicamycin for 2 h at 30°C or with 10% fetal bovine serum for 4 h at 37°C. Untreated controls for each strain were grown for 2 h at 30°C. Total RNA was isolated using a MasterPure Yeast RNA Purification Kit (LGC Biosearch Technologies). DNase treatment was performed using the DNA-free DNA Removal Kit (Invitrogen). The quality of purified RNA samples was assessed by Bioanalyzer RNA analysis (Agilent; performed by the London Regional Genomics Centre) to ensure an RNA integrity number (RIN) of 8 or higher.

### RNA sequencing

Library preparation and RNA sequencing analysis were conducted by Azenta Life Sciences using an Illumina HiSeq sequencer. The final concentration of total RNA used for library preparation ranged from 37.3 to 98.5 ng/μl (1.12–2.96 μg). Total RNA was purified using polyA selection and RNA samples were then converted into Illumina TruSeq cDNA libraries. Each sample yielded between 19.4 to 30.1 million 150 bp paired-end sequencing reads. Raw data in FASTQ format and normalized counts were deposited in NCBI Gene Expression Omnibus (Edgar et al., 2002) and are accessible through GEO Series accession number GSE308581.

Quality control and trimming was performed using Trim Galore, a wrapper tool around CutAdapt (Martin, 2011) and FastQC (https://www.bioinformatics.babraham.ac.uk/projects/fastqc/). Trim Galore default settings were used to remove Illumina adapter sequences and to remove any reads with a length below 50 bp. Reads were then aligned to the reference genome of *Candida albicans* SC5314 Assembly 22 (NCBI RefSeq assembly GCF_000182965.3; GenBank assembly GCA_000182965.3) using STAR (Dobin et al., 2013). FeatureCounts was used to count the reads mapped to each gene (Liao et al., 2014). Differential expression analysis was performed with DESeq2 (Love et al., 2014). Independent hypothesis weighting was performed with an alpha value of 0.1 and using a comparison that takes into account both strain and treatment (Bourgon et al., 2010). An interaction was added to the model to test if the effect of treatment differs according to strain. K-means clustering based on Euclidean distance was used to cluster significant transcripts in nine and seven groups for the tunicamycin and morphogenesis dataset, respectively (Tables S4 and S5). Heat maps were generated using only genes with a *P*_adj_<0.05 (*n*=3429 genes for tunicamycin dataset; *n*=3480 genes for morphogenesis dataset) (Anders and Huber, 2010). Gene ontology analysis for biological processes was performed using ViSEAGO (Brionne et al., 2019). Gene ontology analysis for cellular compartments (morphogenesis dataset) was performed with GO Enrichment Analysis, powered by PANTHER (Ashburner et al., 2000; Gene Ontology Consortium et al., 2023; Thomas et al., 2022) (Tables S6–S8). Data for principal component analysis plots underwent variance stabilizing transformation.

### Hit validation and GRACE library

The two clusters containing genes upregulated in an Ire1- and tunicamycin- or serum+37°C-dependent manner (cluster 2 and 6, respectively) were selected, and the top differentially expressed genes from these clusters were determined. The top differentially expressed genes were then compared to strains available in the GRACE library. Strains in this library contain mutations that enable a specific gene to be repressed upon treatment with doxycycline (Roemer et al., 2003). Only genes with mutants available in the GRACE library or its expansion were selected (Fu et al., 2021; Roemer et al., 2003). A mini library of 21 strains (tunicamycin) and 20 strains (serum) were constructed (Table S9). Each contains CaSS1 as a control strain, and each strain was represented in the library in quadruplicate. *HAC1* was added as an additional strain to the tunicamycin library; it was not in the top 20 genes but was of interest given its role in the UPR.

For the tunicamycin mini-library, strains were spotted into liquid YPD and grown overnight at 30°C. Next, the strains were spotted into YPD, YPD+0.05 μg/ml doxycycline or YPD+20 μg/ml doxycycline for induction and grown overnight at 30°C. The lower dose and higher dose of doxycycline induce gene repression for essential and non-essential genes, respectively. The following day, strains from each condition were spotted into YPD medium containing the same doxycycline treatment with or without 1.5 μg/ml tunicamycin and grown overnight at 30°C. Plates were then mixed briefly prior to measuring the endpoint OD_600_. Data was analyzed as described by Lee et al. (2022). Median absolute deviation (MAD) was calculated for the −tunicamycin and +tunicamycin conditions and plotted. Genes in the lower left and right quadrants were identified as hits (Lee et al., 2022). Several genes falling slightly outside of these quadrants were still considered as genes of interest.

For the top 20 genes from morphogenesis cluster 6, the PathoYeastrack Rank Genes by Transcription Factor function was used to search for transcription factors that regulate the group of 20 genes (Teixeira et al., 2023). The search criteria for this included: documented regulations were filtered by DNA binding or expression evidence, and all expression evidence was included (TF acting as activator, TF acting as inhibitor, and regulations without association information). All transcription factors were checked for, and no other criteria were specified. The full table of results is reported in Table S9. Transcription factors were categorized as regulating morphogenesis, biofilm formation, adhesion, or another function based on GO annotations and mutant phenotype data available in the CDG (Lew-Smith et al., 2025).

### RT-qPCR

Overnight cell cultures were grown in triplicate or quadruplicate. In the morning, cultures were diluted 1:10 in appropriate medium and grown for 90 min to log phase, then treated with 1.5 μg/ml tunicamycin for an additional 2 h at 30°C or with 10% fetal bovine serum for an additional 4 h at 37°C. Untreated cells were grown for an additional 2 h at 37°C to serve as a control. RNA purification and DNase treatment were performed as described above. cDNA libraries were prepared from 2.0–2.5 μg of total RNA with the SuperScript IV VILO Master Mix (Thermo Fisher Scientific) according to the manufacturer's instructions. RT-qPCR was performed using the SYBR Green Master Mix (Thermo Fisher Scientific). Primers used for RT-qPCR can be found in Table S2. *ACT1* was used as a housekeeping gene for all *C. albicans* genes, and RT-qPCR was performed on a QuantStudio 3 real-time PCR system (Thermo Fisher Scientific) and data were analyzed using the comparative Ct method (Schmittgen and Livak, 2008). Unless otherwise stated, all ΔCt values were normalized to the ΔCt of the untreated control strain to obtain ΔΔCt values. Statistical analysis was performed in either IBM SPSS Statistics or GraphPad Prism.

### *HAC1* splicing

Overnight DAY286 cultures were diluted to OD_600_ 0.2 and grown to log phase. Tunicamycin and fetal bovine serum treatments were administered as described in the RNA purification section above. For the splicing time course with serum, cells were immediately removed after addition of serum to serve as the 0 min timepoint. Additional aliquots of cells were removed at the indicated timepoints post-treatment, after which they were washed with cold water and frozen until RNA purification.

RNA purification and DNase treatment were performed as described above. RT-PCR was performed using *HAC1* splicing primers (Table S2) with the SuperScript IV One-Step RT-PCR System (Thermo Fisher Scientific). Samples were run on 4% agarose gels and imaged using a Gel Doc XR+ Imager (Bio-Rad). Gel quantification was performed following the methods described in Petropavlovskiy et al. (2020) to obtain mean grey values (MGVs) for each band. The MGV ratio of spliced *HAC1* to total *HAC1* was determined, and one-way ANOVA with Tukey's multiple comparisons test was performed using IBM SPSS Statistics. *HAC1* splicing was also detected using RT-qPCR with primers that bind to a region in the open reading frame (ORF) of both spliced and unspliced *HAC1* (total *HAC1* primers) and primers that only bind if the *HAC1* intron is removed (spliced *HAC1* primers; Table S2). Statistical analysis was performed in GraphPad Prism. Full uncropped images of gels in this paper are shown in Fig. S12.

### Ire1 secretory transcript target determination and motif search

This protocol was based on analysis performed in Kimmig et al. (2012), for *S. pombe*. For all genes detected in our RNA sequencing (*n*>6000 genes), the effect of strain in the treated samples (tunicamycin or serum+37°C), and the treatment effect in the wild-type strain were calculated. Genes with fold changes with *P*_adj_<0.05 for both variables were plotted and considered for further analysis (Fig. 6A,B; *n*=2685 genes tunicamycin, *n*=1773 genes morphogenesis). For the tunicamycin treatment, 30 genes with a decrease in expression greater than 1.5-fold associated with both strain and tunicamycin treatment were considered (Table S10). For the serum+37°C treatment, 23 genes with a decrease in expression greater than 1.0-fold associated with both strain and serum+37°C were considered (Table S11). The CDG was used to determine protein product, cell component and presence of a transmembrane domain for each gene (Lew-Smith et al., 2025). The CGD definition of cell surface is any protein localized to the external part of the cell wall and/or plasma membrane.

For STREME analysis, the full *C. albicans* Assembly 22 coding sequence was obtained from the CDG and used as a control set (Lew-Smith et al., 2025). Coding sequences for each of the 30 tunicamycin genes and 23 morphogenesis genes were also obtained from CGD. Motifs were restricted to a minimum width of 4 and maximum width of 10. No other settings were changed from the default. For Tomtom motif comparison, the shared motif of interest (ACTGCTGCT) was compared to the JASPAR2026_CORE_fungi_redundant database (Tables S12 and S13).

### Transcriptional inhibition with thiolutin

Overnight cell cultures were grown in triplicate. In the morning, cultures were diluted 1:10 and grown in appropriate media for 90 min, then treated with 1.5 μg/ml tunicamycin and/or 10 μg/ml thiolutin. 1.5 ml of cells were harvested immediately (0) or at 60 or 120 min after treatment. RNA purification, DNase treatment, cDNA library synthesis and RT-qPCR were performed as described above. Each timepoint is normalized to the zero-minute timepoint of the same condition.

## Supplementary Material



10.1242/joces.264610_sup1 Supplementary information

Table S1. All strains used in this study.

Table S2. All plasmids and primers used in this study.

Table S3. All oligos used in this study.

Table S4. Tunicamycin RNA sequencing processed data

Table S5. Morphogenesis RNA sequencing data

Table S6. Tunicamycin RNA sequencing Cluster 2 and Gene Ontology analysis for biological processes

Table S7. Morphogenesis Cluster 6 and Gene Ontology analysis for Biological Processess & Cellular Compartment

Table S8. Cluster 2 (tunicamycin) vs. Cluster 6 (morphogenesis) - Genes in Common & their Gene Ontology terms

Table S9. Top Hit Mini Libraries

Table S10. Protein product, cell component, and TM domain checked with Candida Genome Database

Table S11. Protein product, cell component, and TM domain checked with Candida Genome Database

Table S12. TUNICAMYCIN

Table S13.
