# Supplementary material for: Hac1-independent functions of Ire1 drive morphogenesis in Candida albicans through distinct transcriptional programs
Source: J Cell Sci. 2026 Jul 21;139(14):jcs264610. doi: 10.1242/jcs.264610 (PMC13450900; doi:10.1242/jcs.264610)
Supplement: Supplementary file 1 [file joces-139-264610-s1.pdf]

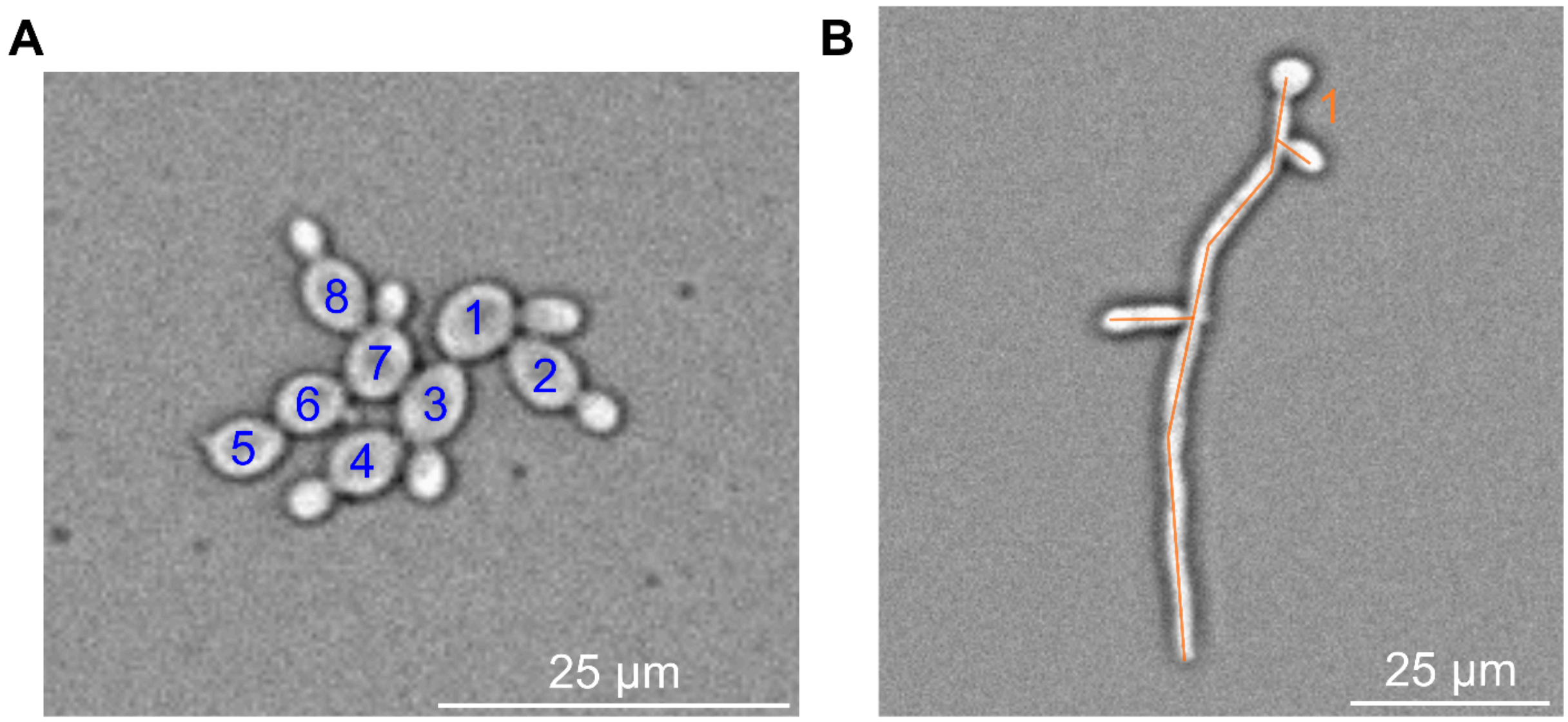

Fig. S1. Filament quantification method. Cells were classified as round or filamentous for the quantification of percent of cells filamenting. **A.** Round cells, numbered in blue. Some round cells have buds, which are not considered unique cells during counting. **B.** Filamentous cell, outlined in orange.

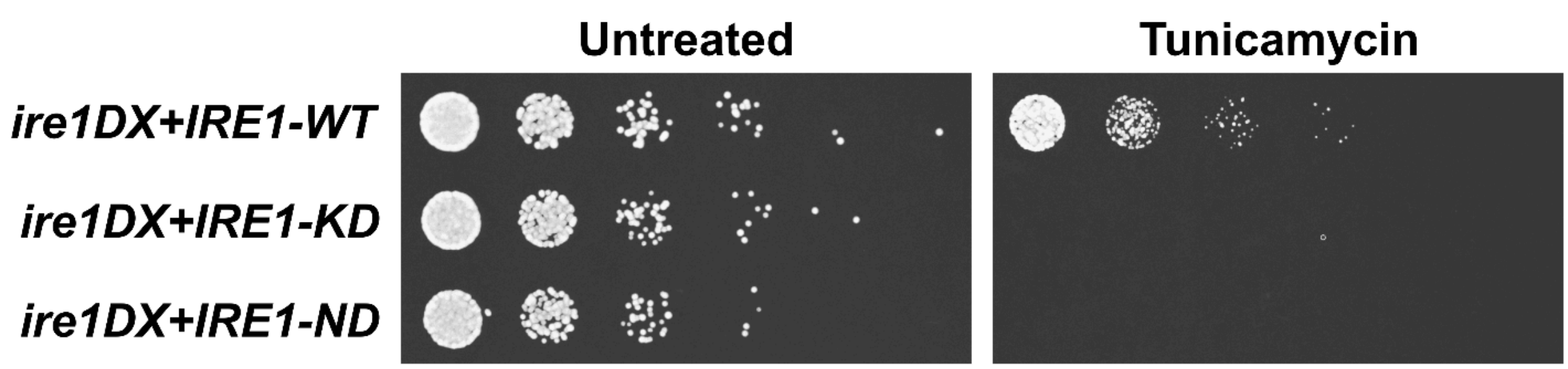

Fig. S2. Ire1 kinase and nuclease domains are required for growth in the presence of tunicamycin. *C. albicans ire1DX* cells complemented with wild-type (WT) *IRE1*, kinase-dead (KD) *IRE1*, and nuclease-dead (ND) *IRE1* were grown on agar plates containing 1.5 µg/mL tunicamycin. Images were acquired after 24 hours.

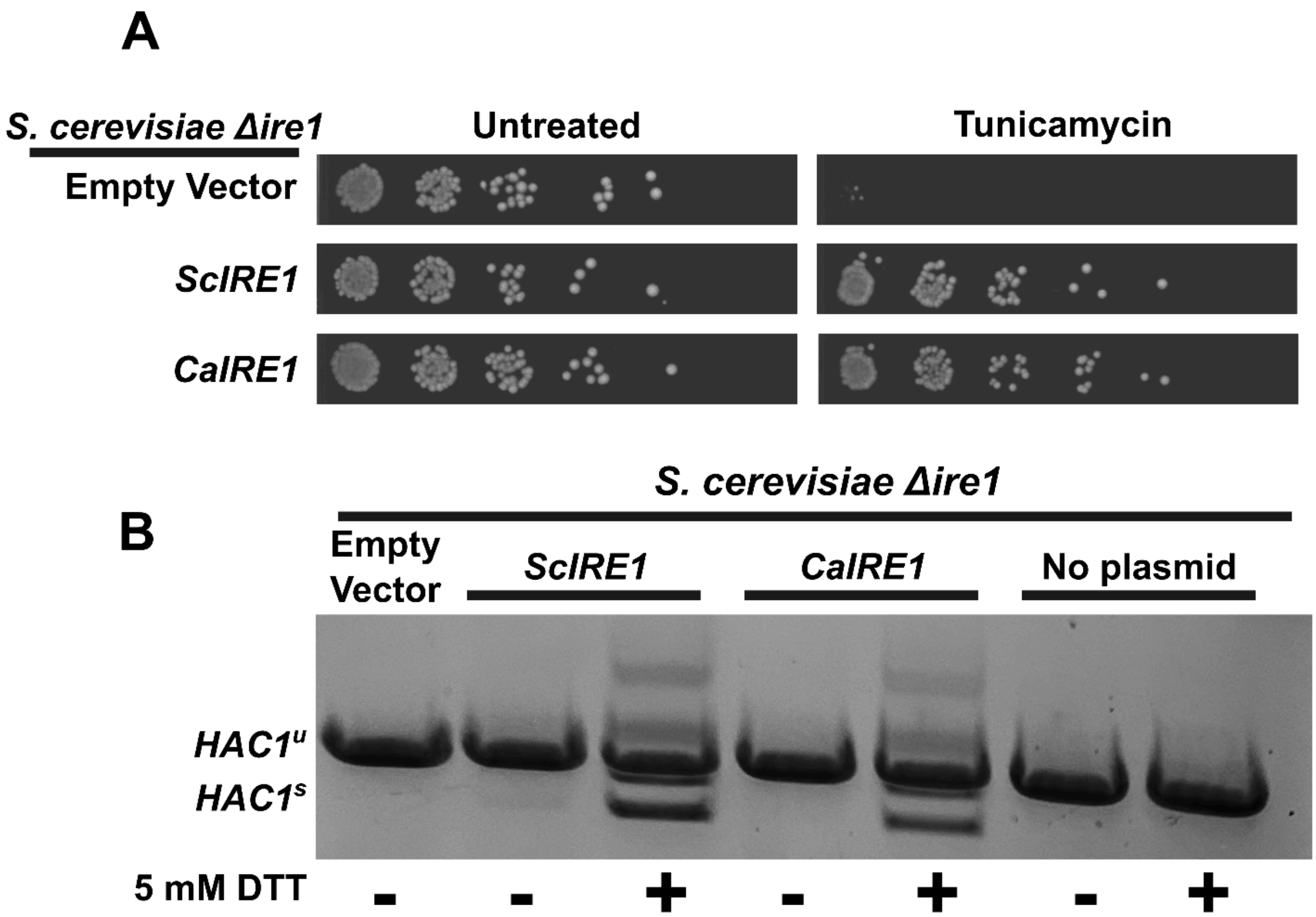

Fig. S3. *C. albicans IRE1* complements the *IRE1* function of *S. cerevisiae*. A. *S. cerevisiae ire1Δ* cells containing the indicated plasmids were grown on agar plates with 1.5 µg/mL tunicamycin. The empty vector is *pRS313*, *ScIRE1* is *pRS313-ScIRE1*, and *CaIRE1* is *pRS313-ScPromoter-CaIRE1*. Images were acquired after 48 hours. **B.** The same strains were treated with 5 mM DTT for 40 minutes, and total RNA was purified. RT-PCR was performed using primers flanking the *HAC1* intron, and the RT-PCR products were resolved on a 1% agarose gel.

Journal of Cell Science • Supplementary information

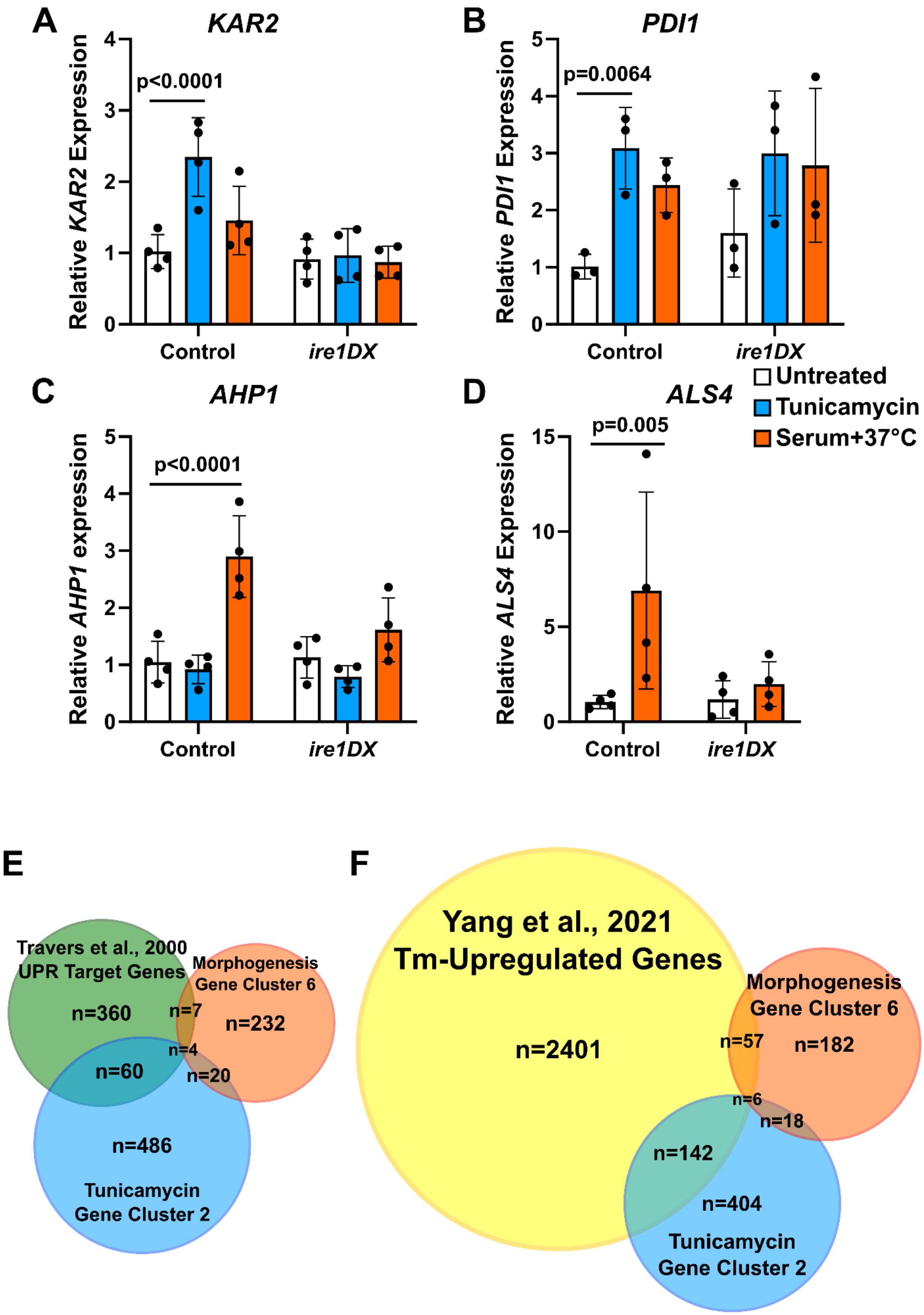

Journal of Cell Science • Supplementary information

Fig. S4. RT-qPCR validation of RNA sequencing hits. Relative gene expression for **A.** *KAR2*, **B.** *PDI1*, **C.** *AHP1*, and **D.** *ALS4* in control (DAY286) and *ire1DX* strains. Mean$\pm$SD shown. One-way ANOVA with Tukey's multiple comparisons test was performed. n=3 for *PDI1* and n=4 for *KAR2, AHP1*, and *ALS4*. Legend applies to all panels . **E.** Comparison of genes in tunicamycin cluster 2 and morphogenesis cluster 6 with *S. cerevisiae* UPR target genes identified by Travers et al.,2000. **F.** Comparison with *C. albicans* tunicamycin-induced genes (1 µg/mL) identified by Yang et al., 2021.

Journal of Cell Science • Supplementary information

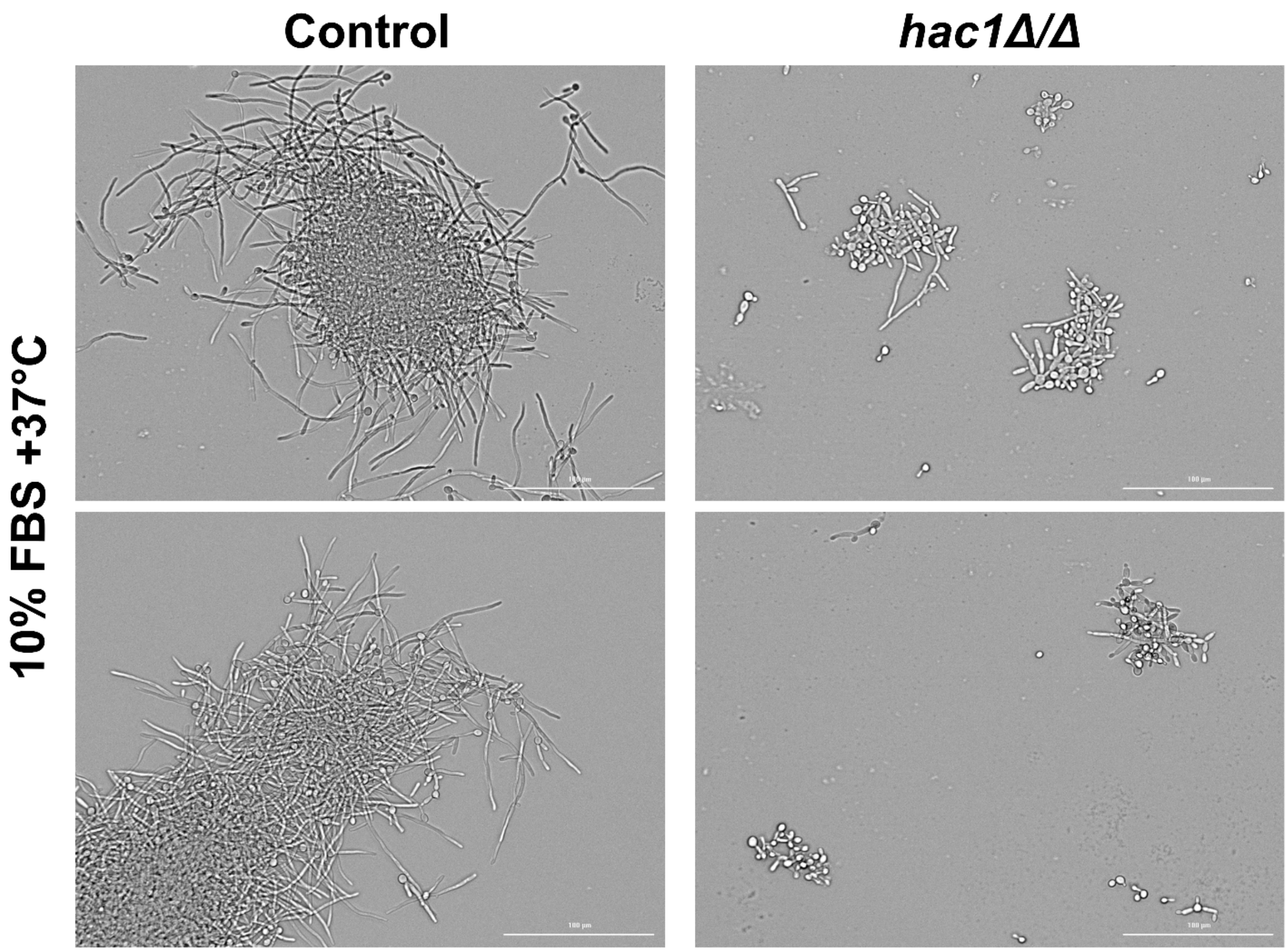

Fig. S5. *C. albicans* cells lacking *HAC1* do not form large flocs. Control (DAY286-Cas9) and *hac1Δ/Δ* strains were grown in filamentation-inducing 10% fetal bovine serum (FBS) for four hours at 37°C. Images obtained using the BioTek Cytation 5 Cell Imaging Multimode Reader. Scale bar = 100 µm.

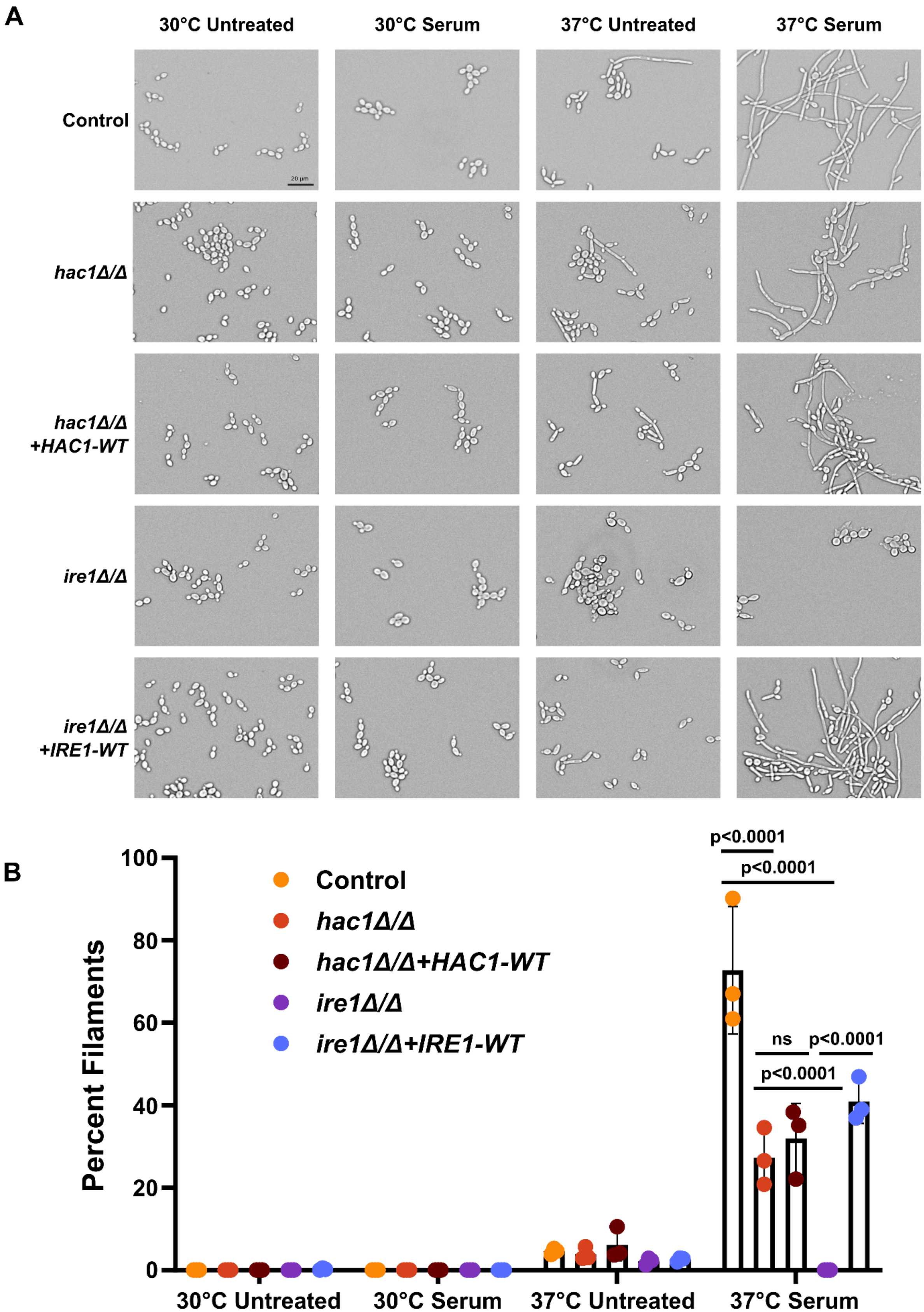

Journal of Cell Science • Supplementary information

Fig. S6. *HAC1* is not required for morphogenesis. A. Control (SC5314), *hac1Δ/Δ*, *hac1Δ/Δ +HAC1-WT, ire1Δ/Δ, and ire1Δ/Δ+IRE1-WT C. albicans* cells were grown for four hours either in filamentation-inducing 10% fetal bovine serum at 37°C or under untreated control conditions at 30°C. Images obtained using the BioTek Cytation 5 Cell Imaging Multimode Reader. Scale bar = 20 μm, applies to all images. **B.** Filamentation was quantified for 3 biological replicates. Photos were acquired from randomly selected portions of each slide, and round and filamentous cells were counted by hand for each photo. Percent filaments was calculated and mean±SD was plotted.

Journal of Cell Science • Supplementary information

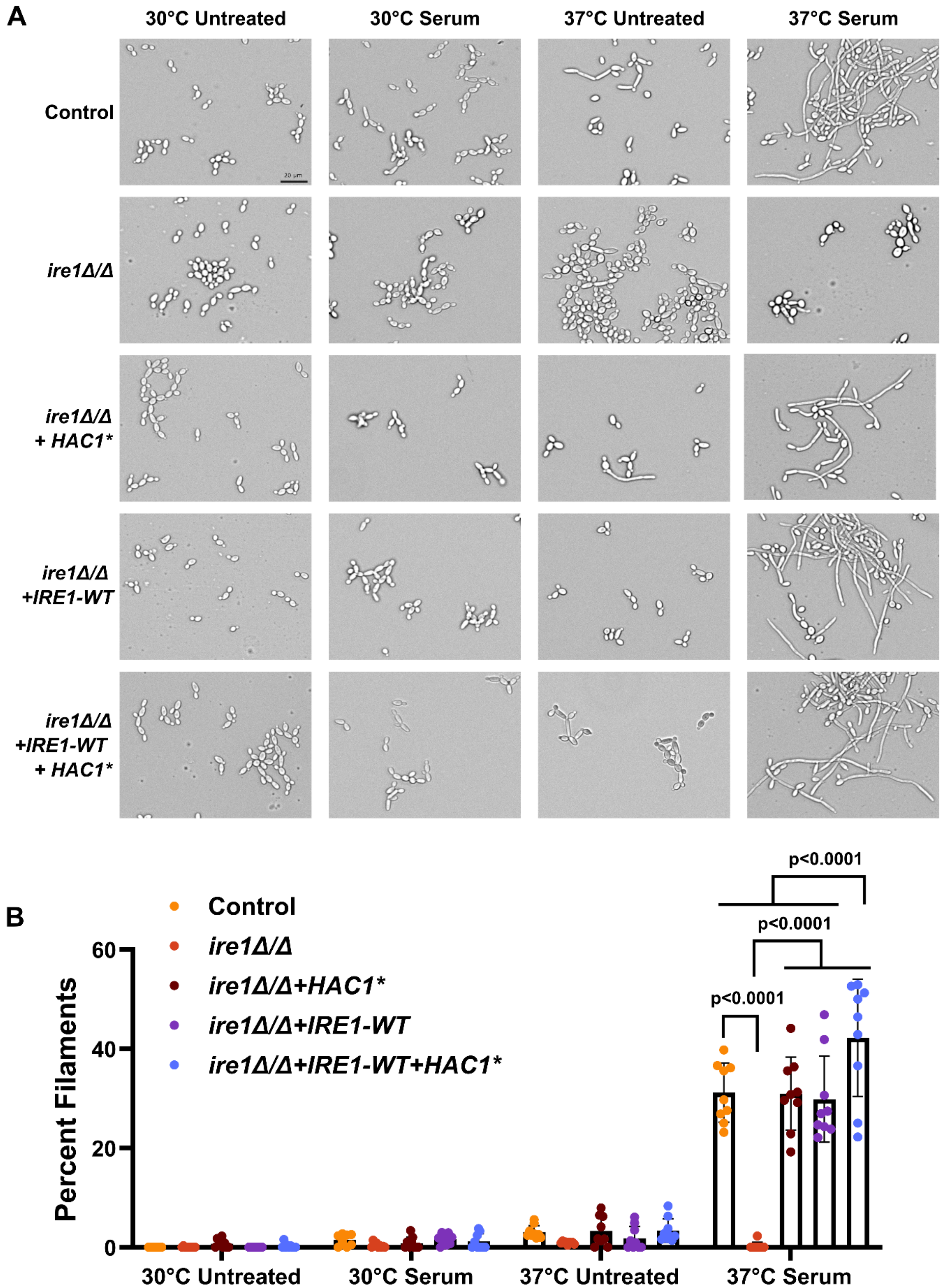
A
30°C Untreated
30°C Serum
37°C Untreated
37°C Serum
Control
ire1Δ/Δ
ire1Δ/Δ + HAC1*
ire1Δ/Δ +IRE1-WT
ire1Δ/Δ +IRE1-WT + HAC1*
B
Control
ire1Δ/Δ
ire1Δ/Δ+HAC1*
ire1Δ/Δ+IRE1-WT
ire1Δ/Δ+IRE1-WT+HAC1*
Percent Filaments
60
40
20
0
p<0.0001
p<0.0001
p<0.0001
30°C Untreated
30°C Serum
37°C Untreated
37°C Serum

Fig. S7. Constitutively spliced *HAC1* recovers filamentation in the absence of *IRE1*. A. Control (SC5314), *ire1Δ/Δ, ire1Δ/Δ+IRE1-WT, ire1Δ/Δ+HAC1* (*constitutively spliced *HAC1),* and *ire1Δ/Δ+IRE1-WT+HAC1* C. albicans* cells were grown for four hours either in filamentation-inducing 10% fetal bovine serum at 37°C or under untreated control conditions at 30°C. Images obtained using the BioTek Cytation 5 Cell Imaging Multimode Reader. Scale bar = 20 μm, applies to all images. **B.** Filamentation was quantified for 3 biological replicates. Photos were acquired from randomly selected portions of each slide, and round and filamentous cells were counted by hand for each photo. Percent filaments was calculated, and mean±SD was plotted.

Journal of Cell Science • Supplementary information

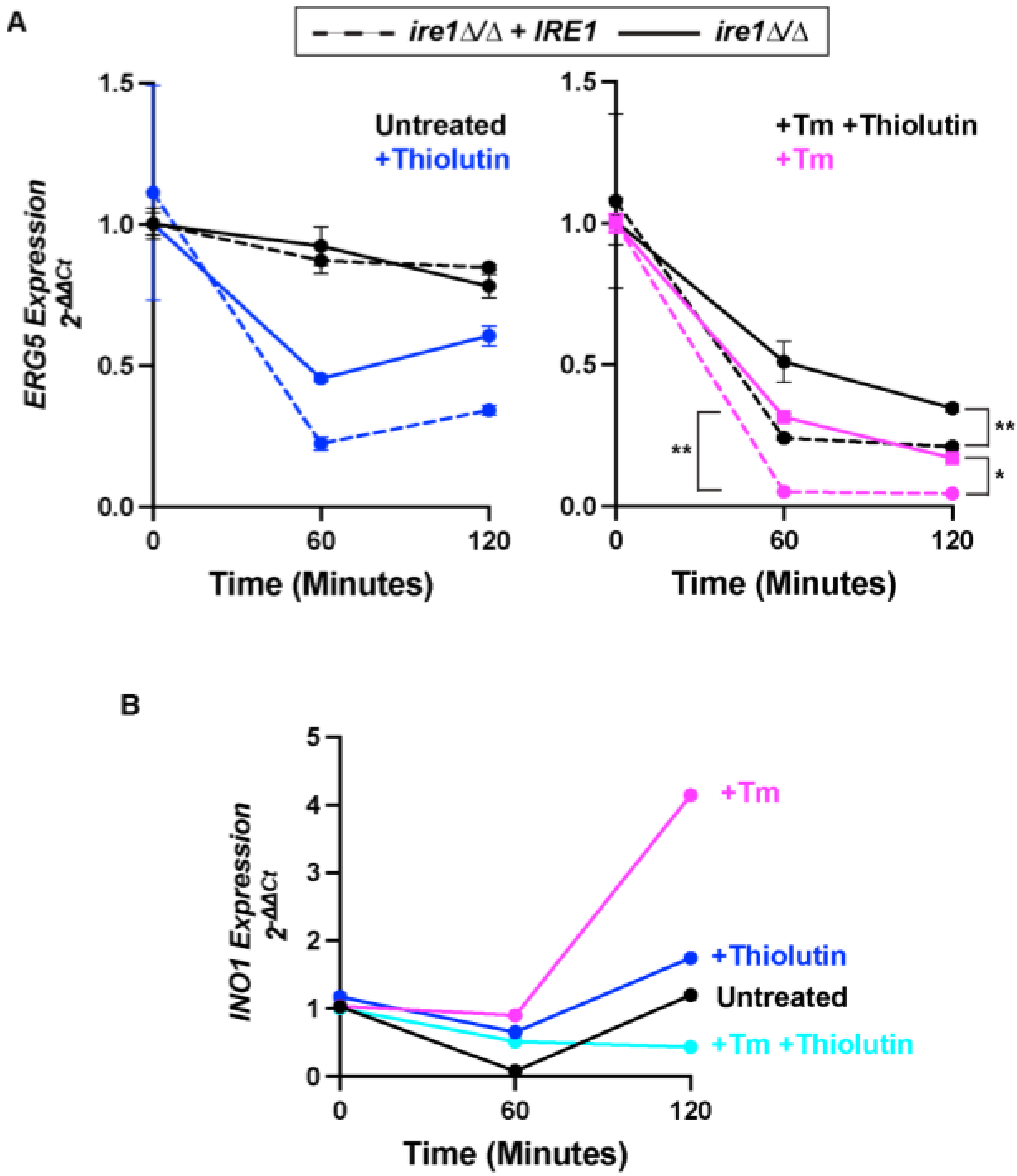

Fig. S8. Transcriptional inhibition with thiolutin. A. Relative gene expression for *ERG5* in *ire1Δ/Δ+IRE1-WT* (dashed lines) and *ire1Δ/Δ* (solid lines) strains. Left panel: untreated (black) and thiolutin-treated (blue) conditions. Right panel: tunicamycin (pink) and tunicamycin+thiolutin (black) conditions. T he x-axis represents time post-treatment. Each timepoint is normalized to

Journal of Cell Science • Supplementary information

the zero-minute timepoint of the same condition, mean±SE shown. *p<0.05 **p<0.01 by two-way ANOVA. **B.** Relative gene expression for *INO1* in control (SC5314) cells treated with tunicamycin (pink), thiolutin (blue), tunicamycin+thiolutin (cyan), or untreated (black). The increase in *INO1* expression with thiolutin alone suggests that thiolutin may induce ER stress responses. Each timepoint is normalized to the zero minute timepoint of the same condition, mean±SE shown.

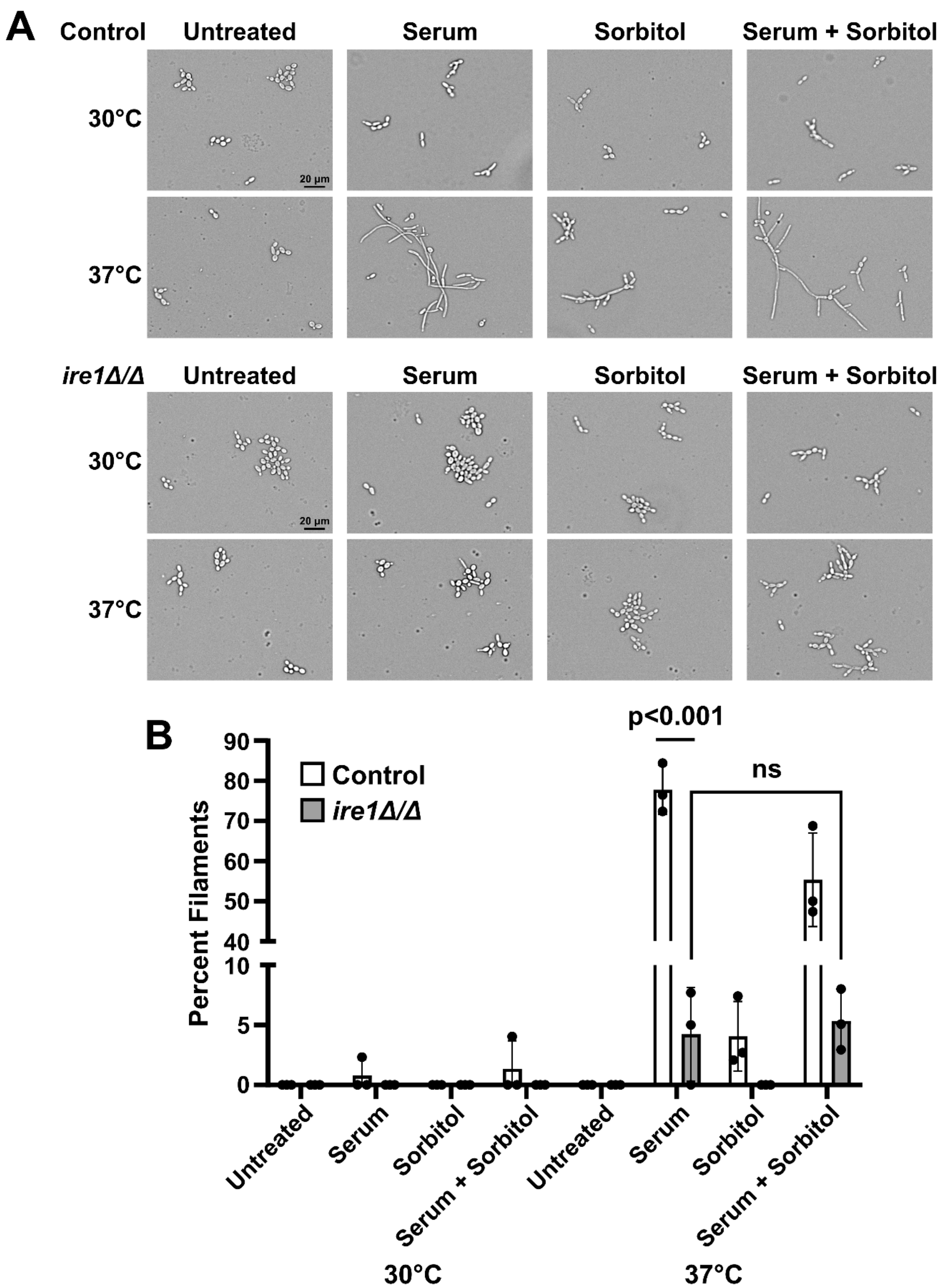

Journal of Cell Science • Supplementary information

Fig. S9. Sorbitol does not rescue morphogenesis in *IRE1*-deficient cells. A. DAY286-Cas9 (control) and *ire1Δ/Δ* cells were grown for four hours in filamentation-inducing 10% fetal bovine serum with or without 1 M sorbitol for four hours at 37°C, with untreated cells grown at 30°C as a control. Images obtained using the BioTek Cytation 5 Cell Imaging Multimode Reader. Scale bar = 20 µm, applies to all images. **B.** Filamentation was quantified using photos acquired from randomly selected portions of each slide, and round and filamentous cells were counted by hand for each photo. Percent filaments was calculated, and mean±SD was plotted, n=3.

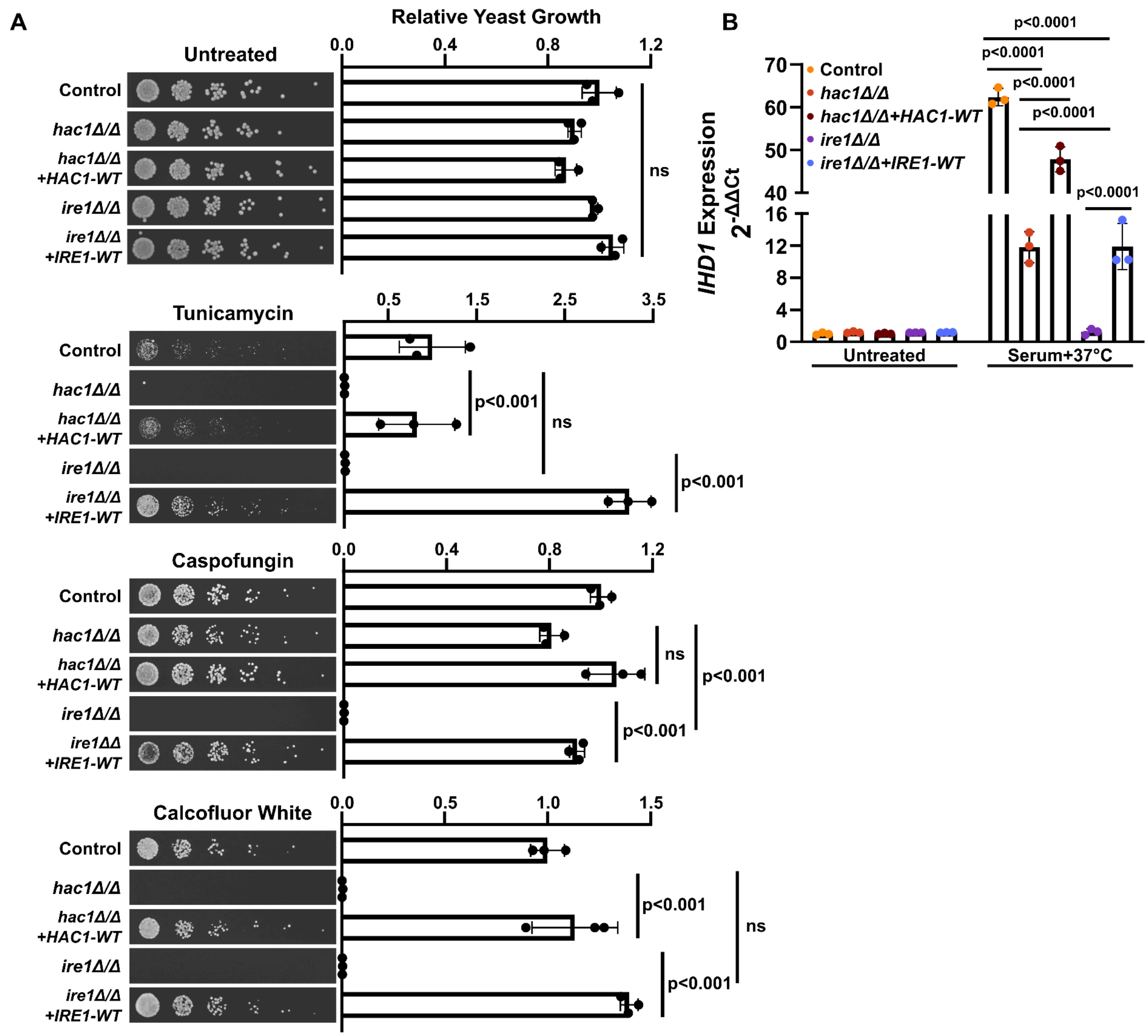

Fig. S10.. *IRE1* contributes more strongly to cell wall stress tolerance than *HAC1*. A. Control (SC5314), *hac1Δ/Δ*, *hac1Δ/Δ+HAC1-WT, ire1Δ/Δ, and ire1Δ/Δ+IRE1-WT C. albicans* cells were spotted on plates containing 1.5 µg/mL tunicamycin, 0.02 µg/mL caspofungin, and 10 µg/mL calcofluor white. Images were acquired after 24 hours. Statistical analysis was performed using One-way ANOVA followed by Tukey's multiple comparisons test, n=3, mean ±SD plotted. **B.** Relative gene expression for *IHD1* in indicated strains, SC5314 was used as the control strain. Mean±SD shown. One-way ANOVA with Tukey's multiple comparisons test was performed, n=3

Journal of Cell Science • Supplementary information

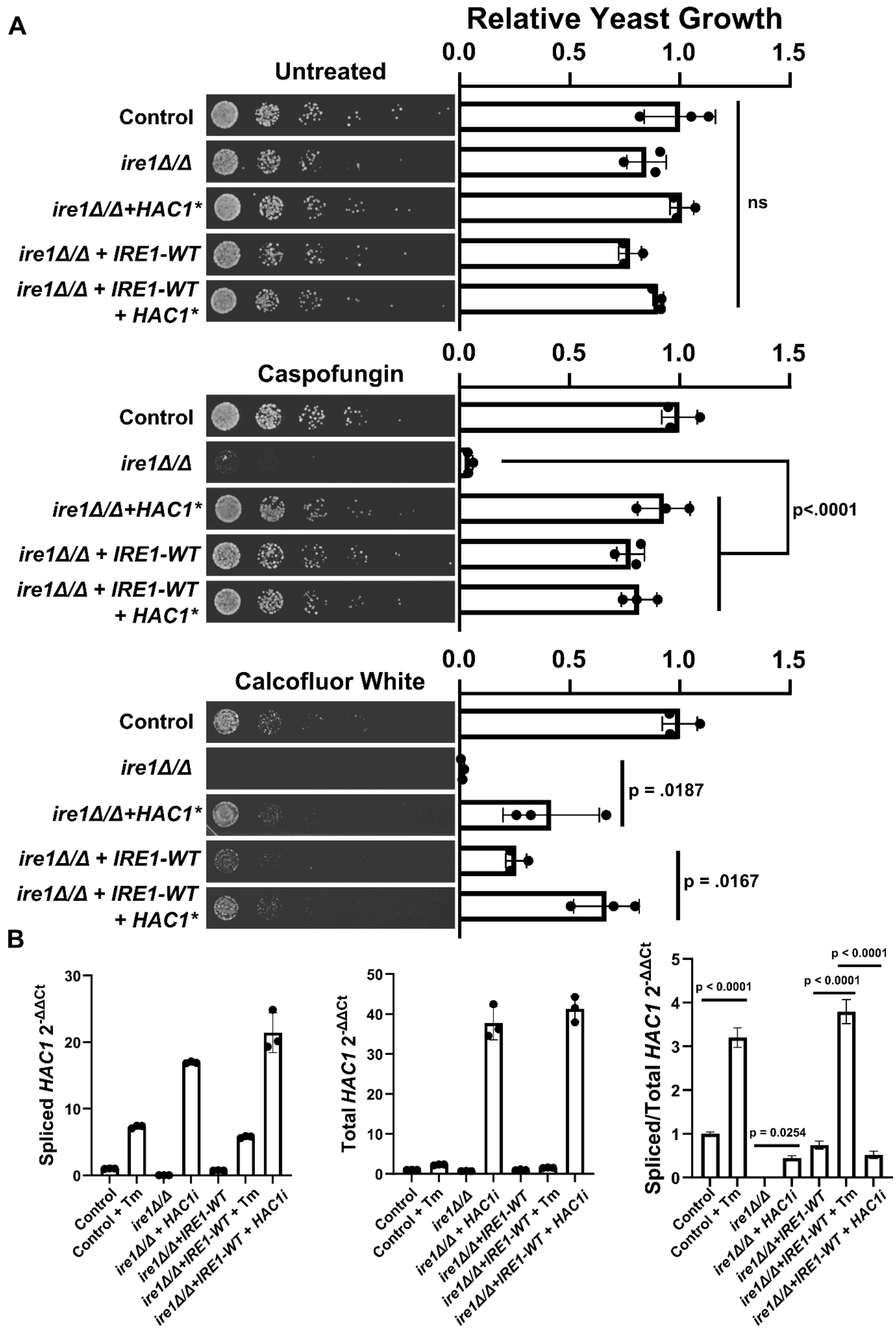

Journal of Cell Science • Supplementary information

Fig. S11. Constitutively spliced *HAC1 rescues growth of *IRE1* deletion strain in the presence of cell wall stressors. A.** Control (SC5314), *ire1Δ/Δ, ire1Δ/Δ+IRE1-WT, ire1Δ/Δ +HAC1* (*constitutively spliced *HAC1),* and *ire1Δ/Δ+IRE1-WT+HAC1* C. albicans* cells were spotted on plates containing 0.01 μg/mL caspofungin and 10 μg/mL calcofluor white. Images were acquired after 24 hours. Statistical analysis performed using a One-way ANOVA with Tukey's multiple comparisons test, n=3, mean±SD plotted. **B.** Relative gene expression for spliced *HAC1*, total *HAC1*, and spliced: total *HAC1* ratio in the indicated strains. Where indicated, tunicamycin was added at 1.5 μg/mL. SC5314 was used as the control strain. One-way ANOVA with Tukey's multiple comparisons test was performed, n=3.

Figure 5C,D Gels

Untreated *HAC1* splicing

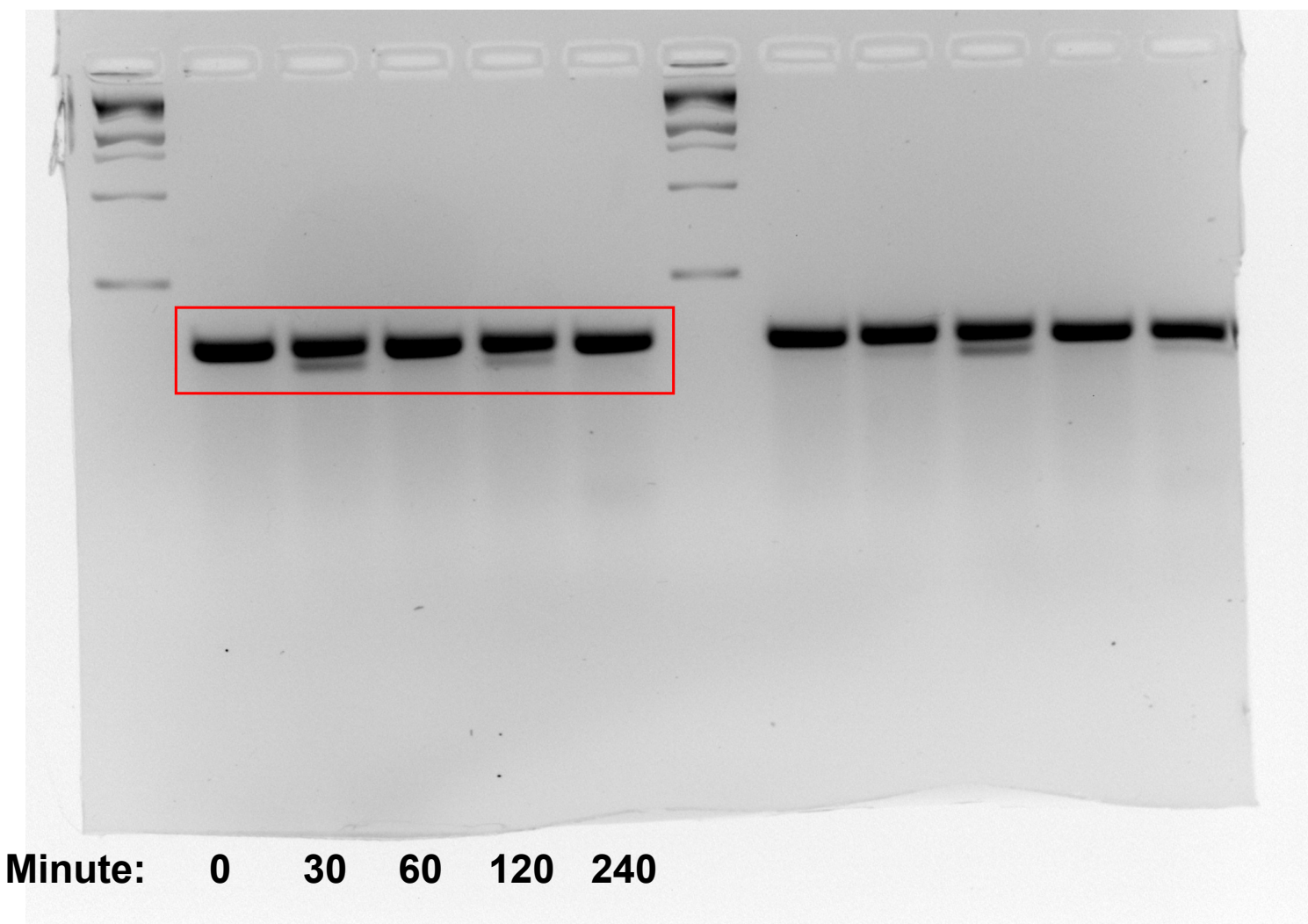

Serum+37C *HAC1* splicing

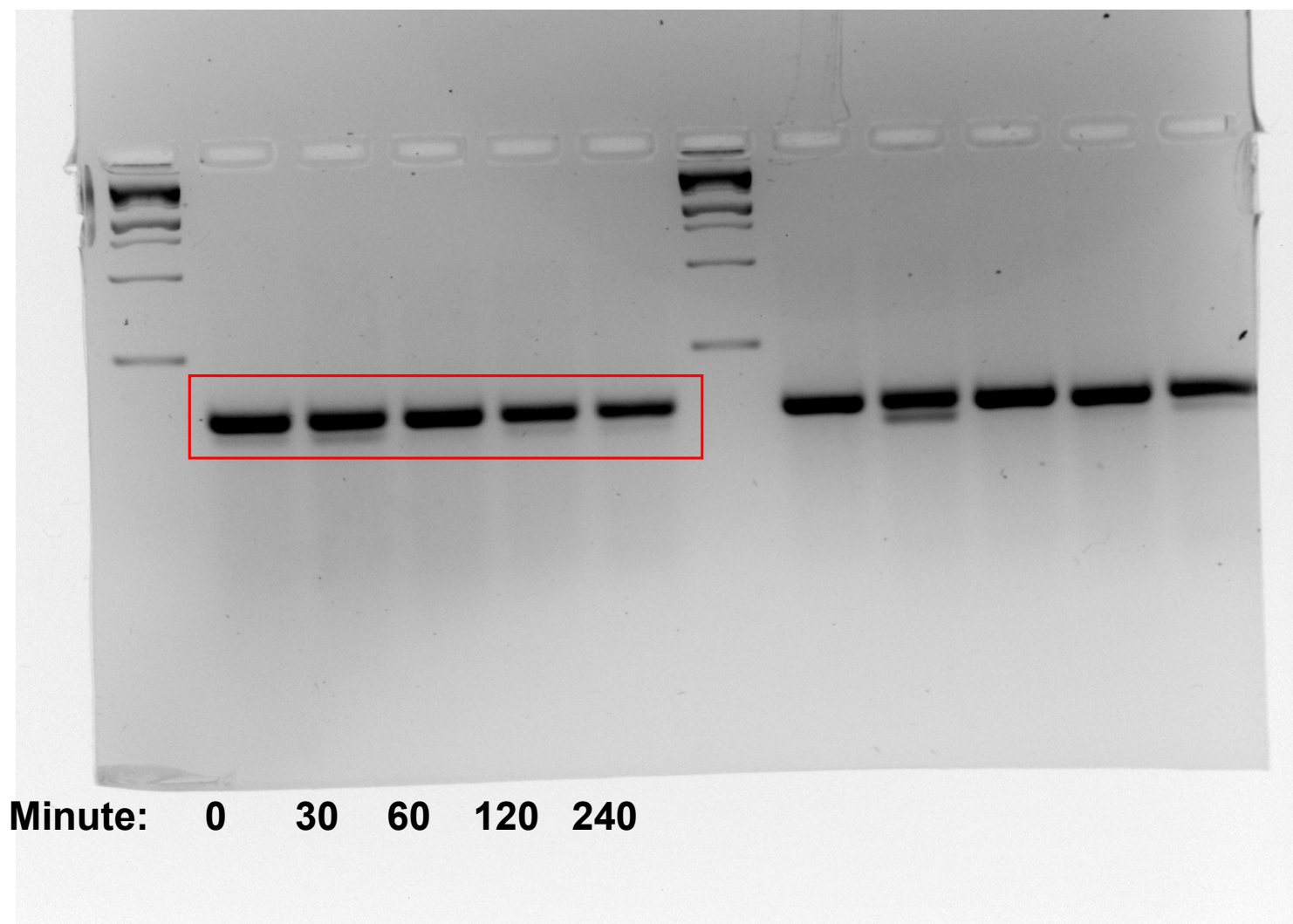

Tunicamycin *HAC1* splicing

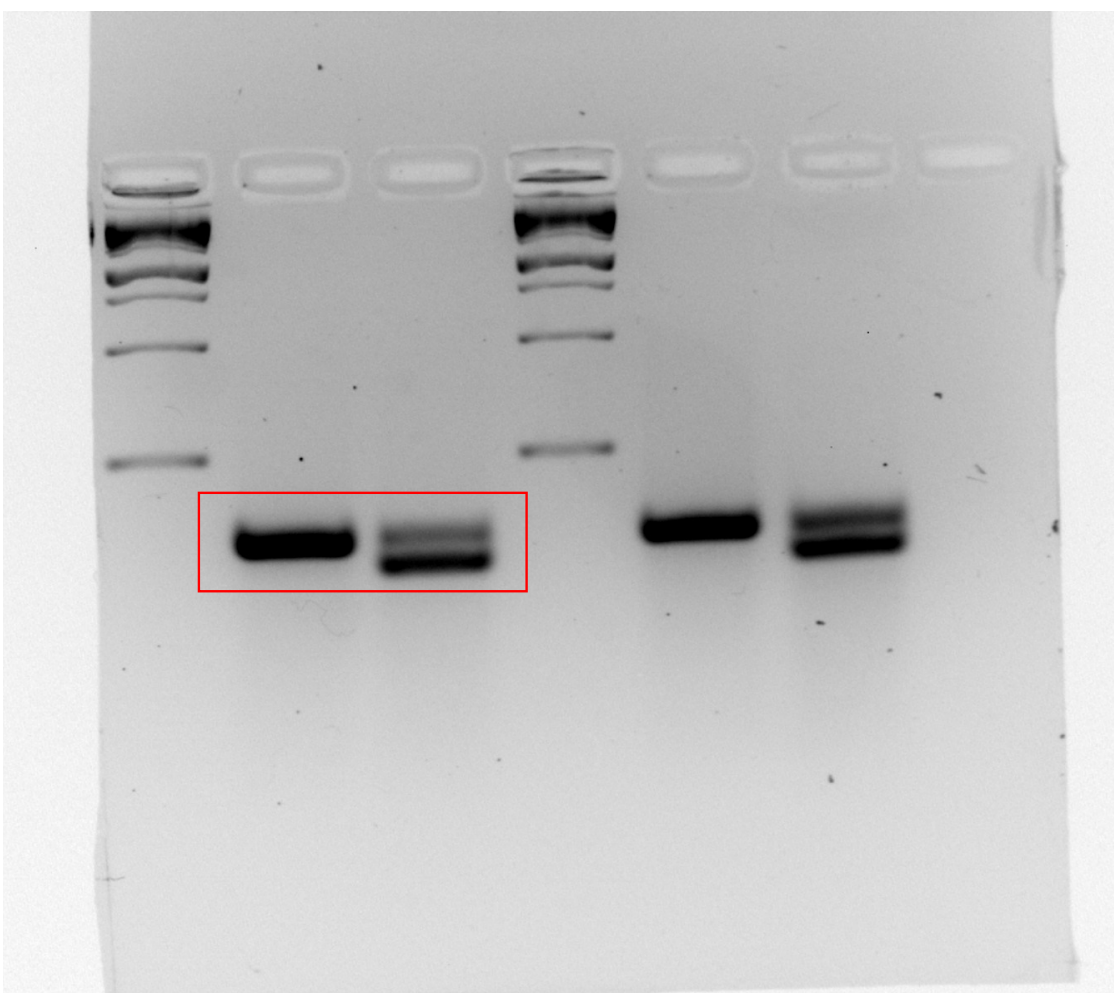

Minute: 0 120

Figure 1C Gel

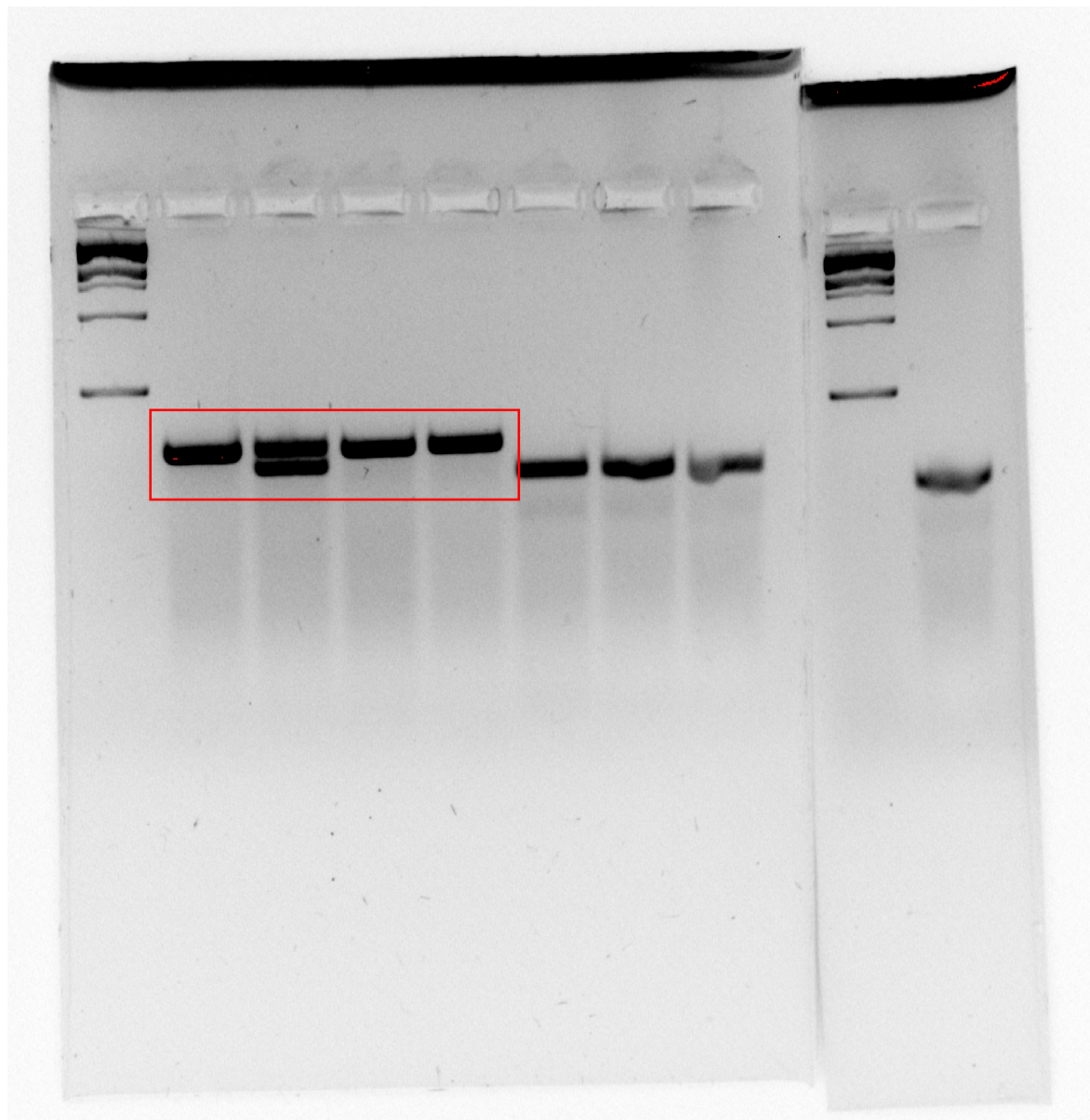

Supp Fig 3B Gel

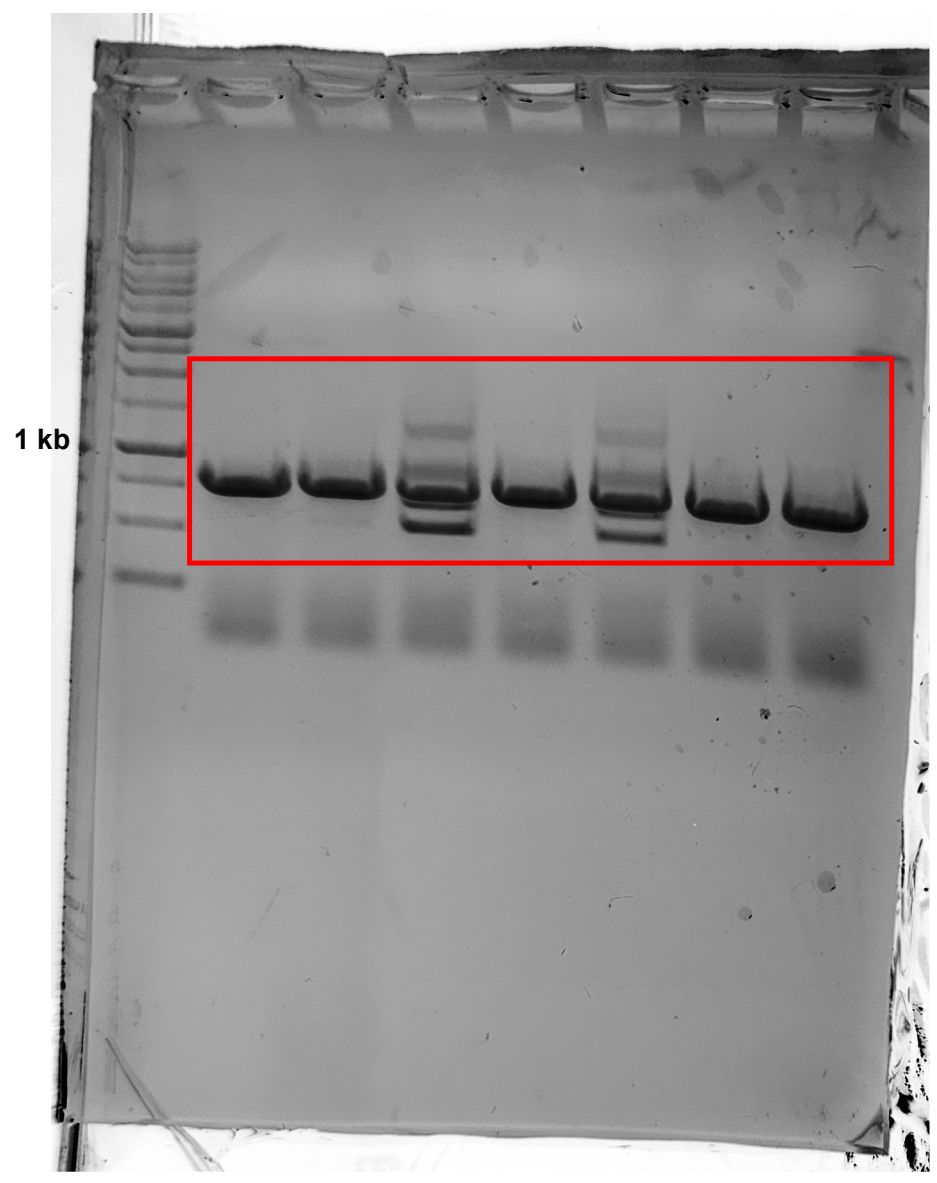

Fig. S12. Gel Transparency. Full gels are shown for the corresponding figures. Red boxes identify regions that were included in figures. All images were acquired using a Gel Doc XR+ Imager (Bio-Rad).

Journal of Cell Science • Supplementary information

Table S1. All strains used in this study.

Available for download at
https://journals.biologists.com/jcs/article-lookup/doi/10.1242/jcs.264610#supplementary-data

Table S2. All plasmids and primers used in this study.

Available for download at
https://journals.biologists.com/jcs/article-lookup/doi/10.1242/jcs.264610#supplementary-data

Table S3. All oligos used in this study.

Available for download at
https://journals.biologists.com/jcs/article-lookup/doi/10.1242/jcs.264610#supplementary-data

Table S4. Tunicamycin RNA sequencing processed data

Available for download at
https://journals.biologists.com/jcs/article-lookup/doi/10.1242/jcs.264610#supplementary-data

Table S5. Morphogenesis RNA sequencing data

Available for download at
https://journals.biologists.com/jcs/article-lookup/doi/10.1242/jcs.264610#supplementary-data

Journal of Cell Science • Supplementary information

Table S6. Tunicamycin RNA sequencing Cluster 2 and Gene Ontology analysis for biological processes

Available for download at
https://journals.biologists.com/jcs/article-lookup/doi/10.1242/jcs.264610#supplementary-data

Table S7. Morphogenesis Cluster 6 and Gene Ontology analysis for Biological Processess & Cellular Compartment

Available for download at
https://journals.biologists.com/jcs/article-lookup/doi/10.1242/jcs.264610#supplementary-data

Table S8. Cluster 2 (tunicamycin) vs. Cluster 6 (morphogenesis) - Genes in Common & their Gene Ontology terms

Available for download at
https://journals.biologists.com/jcs/article-lookup/doi/10.1242/jcs.264610#supplementary-data

Table S9. Top Hit Mini Libraries

Available for download at
https://journals.biologists.com/jcs/article-lookup/doi/10.1242/jcs.264610#supplementary-data

Table S10. Protein product, cell component, and TM domain checked with Candida Genome Database

Available for download at
https://journals.biologists.com/jcs/article-lookup/doi/10.1242/jcs.264610#supplementary-data

Journal of Cell Science • Supplementary information

Table S11. Protein product, cell component, and TM domain checked with Candida Genome Database

Available for download at
https://journals.biologists.com/jcs/article-lookup/doi/10.1242/jcs.264610#supplementary-data

Table S12. TUNICAMYCIN

Available for download at
https://journals.biologists.com/jcs/article-lookup/doi/10.1242/jcs.264610#supplementary-data

Table S13.

Available for download at
https://journals.biologists.com/jcs/article-lookup/doi/10.1242/jcs.264610#supplementary-data

Journal of Cell Science • Supplementary information
