# Supplementary material for: Hac1-independent functions of Ire1 drive morphogenesis in Candida albicans through distinct transcriptional programs
Source: J Cell Sci. 2026 Jul 21;139(14):jcs264610. doi: 10.1242/jcs.264610 (PMC13450900; doi:10.1242/jcs.264610)
Supplement: Supplementary file 2 [file joces-139-264610_review_history.pdf]

Journal of Cell Science

Hac1-independent functions of Ire1 drive morphogenesis in C*andida albicans* through distinct transcriptional programs

Samuel Stack-Couture, Gabriela Nunes Marsiglio Librais, Bryan Lung, Julie Genereaux, Viola Halder, Vanessa Dumeaux, Rebecca S. Shapiro and Patrick Lajoie
DOI: 10.1242/jcs.264610

Editor: Pedro Carvalho

Review timeline

Original submission:	20 November 2025
Editorial decision:	16 December 2025
First revision received:	27 May 2026
Accepted:	10 June 2026

Original submission

First decision letter

MS ID#: jcs.264610

MS TITLE: Distinct Ire1-driven transcriptional responses control morphogenesis in Candida albicans

AUTHORS: Samuel Stack-Couture; Gabriela Librais; Bryan Lung; Julie Genereaux; Viola Halder; Vanessa Dumeaux; Rebecca Shapiro; Patrick Lajoie

ARTICLE TYPE: Research Article

Dear Dr Lajoie,

We have now reached a decision on the above manuscript.

As you will see, the reviewers raise a number of substantial criticisms that prevent me from accepting the paper at this stage. They suggest, however, that a revised version might prove acceptable, if you can address their concerns. In particular, it would be important address major concerns 1 and 3 from reviewer #1 and all other minor concerns. If you think that you can deal satisfactorily with the criticisms on revision, I would be pleased to see a revised manuscript. We would then return it to the reviewers.

Reviewer 1

This manuscript investigates the role of the unfolded protein response (UPR) sensor Ire1 in Candida albicans morphogenesis. The authors propose that Ire1 regulates hyphal development through a transcriptional program that is fundamentally distinct from the canonical, tunicamycin-induced UPR. They suggest this mechanism operates largely independently of the transcription factor Hac1, potentially utilizing regulated Ire1-dependent decay (RIDD) to target secretory pathway transcripts and maintain cell wall integrity during filamentation.

While the study presents an intriguing model for a non-canonical role of Ire1 in morphogenesis, several critical controls and analytical refinements are necessary to support the central claims. The current conclusions are weakened by confounding factors in the transcriptomic analysis, specifically population heterogeneity, and a lack of direct evidence for endonucleolytic decay. To reach the

© 2026. Published by The Company of Biologists under the terms of the Creative Commons Attribution License (https://creativecommons.org/licenses/by/4.0/).

standards for publication, the authors must address these technical gaps, particularly regarding genetic complementation and the distinction between signaling outputs and global secretory dysfunction.

Major Points

1. The RNA-seq analysis comparing genotypes with vastly different hyphal fractions (e.g., ~88% vs ~1%) introduces a major morphotype composition confound. Without normalization for the proportion of hyphae or single-cell resolution, the identified "distinct programs" and RIDD candidates likely reflect population heterogeneity rather than direct Ire1 regulation. The authors should control for this by incorporating hyphal percentage as a covariate or validating key targets in morphotype-matched fractions.

2. The claim of regulated Ire1-dependent decay (RIDD) is currently unsupported by direct evidence of endonucleolytic activity. The data presented relies on steady-state abundance (RNA-seq/qPCR), which cannot distinguish between reduced synthesis and increased decay. To conclusively demonstrate RIDD, the authors must provide evidence of accelerated decay kinetics, map specific cleavage sites, or perform in vitro cleavage assays using the purified Ire1 cytosolic domain and potential RIDD substrates.

3. Critical phenotypes regarding the partial requirement of HAC1 and cell wall stress tolerance rely on deletion strains without matched genetic complementation. Without restoring gene function in the same background, it is impossible to rule out off-target edits or suppressor mutations. Complementation of the full deletion strains is standard practice and essential to validate causality, particularly given the reliance on these strains for the study's genetic arguments. Furthermore, introducing constitutively active spliced HAC1 into the ire1 deletion strain is necessary to fully test the independence of this pathway.

Minor Points

4. The comparison between the canonical UPR (tunicamycin at 30°C) and the morphogenesis program (serum at 37°C) is confounded by differences in temperature and duration. These variables alone can shift gene expression, artificially inflating the apparent divergence between the programs. Temperature-matched controls or time-course analyses are needed to confirm that the "distinctness" is not an artifact of experimental conditions.

5. The conclusion that morphogenesis occurs without HAC1 splicing relies on a gel-based assay that lacks sufficient sensitivity to detect low-amplitude or transient events. As splicing can be subtle during physiological transitions, a more sensitive quantitative method, such as qPCR, is required to definitively rule out minor splicing events.

6. The use of a hypomorphic IRE1 strain for the transcriptomic inference of Ire1 dependence may conflate residual signaling or secondary stress effects with specific Ire1-dependent regulation. Validating key targets using clean deletion and rescue strains in the same experimental design would strengthen the attribution of these gene sets to Ire1 function.

Reviewer 2

It is widely accepted that, in Saccharomycetaceae yeasts, the ER-located transmembrane protein kinase/RNase Ire1 performs splicing of HAC1 mRNA to trigger UPR gene induction to cope with ER stress. In the present study, the authors demonstrated that Ire1 plays a totally distinct function to promote hyphal formation in Candida cells. The data shown here are intriguing and convincing. Moreover, on the whole, the main text is well written. I suggest that the authors revise mainly the discussion section before the acceptance of this paper.

SUGGESTIONS TO AUTHORS

(1) As the authors describe in the manuscript, some previous publications have already suggested Hac1-independent roles of Ire1 in Candida cells. I think that conceptual novelty and importance of

© 2026. Published by The Company of Biologists under the terms of the Creative Commons Attribution License (https://creativecommons.org/licenses/by/4.0/).

the present study should be explained more understandably and clearly to highlight differences from the previous studies.

(2) According to the authors' model shown in Fig. 8, the control of cell wall integrity and the RIDD are two distinct outputs from Ire1. Please explain why authors think so. Alternatively, is it possible that, for instance, cell wall integrity is controlled as a downstream event of the RIDD?

(3) In the Discussion section, please more clearly explain possible mechanism(s) by which Ire1 causes different outputs in response to tunicamycin and to serum+37C. By what mechanism did Ire1 fail to promote HAC1-mRNA splicing when cells were exposed to serum+37C? By what mechanism did Ire1 fail to promote gene expression changes leading to hyphal formation when cells were stressed by tunicamycin?

(4) In the manuscript, it is not clear whether the KD mutant Ire1 has an RNase activity as strong as wild-type Ire1. Autophosphorylation of wild-type Ire1 is a prerequisite for its activation as an RNase. Did the KD mutant exhibit diminished functions simply because its RNase activity was impaired by the KD mutation? Alternatively, can the authors present evidence to claim a role of Ire1's kinase other than autophosphorylation?

(5) As shown in Kimmig et al. (PMID: PMID: 23066505), short fragments of downregulated RNA produced dependently on Ire1 are strong evidence of RIDD. Therefore, I think that a limitation of this study is the lack of data to demonstrate that the downregulated genes shown in Fig 7 are really digested by Ire1. Please address this point in the Discussion section.

First revision

Author response to reviewers' comments

Dear Dr. Carvalho,

Thank you for the opportunity to revise our manuscript. We appreciate the constructive feedback from both reviewers, which has substantially strengthened the paper. In this revision, we have addressed all major and minor concerns raised by both reviewers. The key changes include:

(1) A new paragraph in the Results section directly addressing the morphotype composition confound in our RNA-seq analysis, with three independent lines of evidence supporting our interpretation (Reviewer 1, Major Point 1). (2) New experiments using thiolutin as a transcriptional inhibitor and STREME/Tomtom motif analysis to probe the mechanism of Ire1-dependent transcript reduction, with honest acknowledgment that these approaches were insufficient to confirm RIDD (Reviewer 1, Major Point 2; Reviewer 2, Point 5). (3) Extensive new genetic data using independently generated and previously characterized deletion strains with matched complements (Supp Figs. 6, 7, 10, 11), including constitutively spliced HAC1* in the ire1Δ background, validating all key phenotypes (Reviewer 1, Major Point 3). (4) Sensitive RT-qPCR detection of HAC1 splicing revealing minor, transient splicing during early morphogenesis (Fig. 5E), leading us to revise our conclusion from "no HAC1 splicing" to "limited HAC1 splicing" (Reviewer 1, Minor Point 5). (5) Substantially expanded Discussion sections addressing conceptual novelty (Reviewer 2, Point 1), the relationship between cell wall integrity and secretory transcript degradation (Reviewer 2, Point 2), mechanisms of differential Ire1 signaling with three new references on distinct Ire1 catalytic modes (Reviewer 2, Point 3), and a dedicated paragraph on the limitation of lacking direct cleavage evidence with suggested future experiments (Reviewer 2, Point 5). (6) Careful reframing of RIDD-related claims throughout the manuscript, replacing definitive language with appropriately cautious phrasing such as "Ire1-dependent reduction in transcripts encoding secretory proteins."

We believe these revisions comprehensively address the reviewers' concerns while maintaining the integrity and novelty of our findings. Below we provide point-by-point responses.

© 2026. Published by The Company of Biologists under the terms of the Creative Commons Attribution License (https://creativecommons.org/licenses/by/4.0/).

Reviewer 1 — Major Points

1. The RNA-seq analysis comparing genotypes with vastly different hyphal fractions (e.g., ~88% vs ~1%) introduces a major morphotype composition confound. Without normalization for the proportion of hyphae or single-cell resolution, the identified "distinct programs" and RIDD candidates likely reflect population heterogeneity rather than direct Ire1 regulation. The authors should control for this by incorporating hyphal percentage as a covariate or validating key targets in morphotype-matched fractions.

We appreciate this important concern. We have added a new paragraph in the Results section (beginning with "An inherent challenge in comparing transcriptomes across strains with different filamentation capacities…") that directly addresses the morphotype composition confound with three independent lines of evidence:

First, canonical UPR target genes (*KAR2*, *PDI1*, *HAC1*) show no significant Ire1-dependent fold change under serum+37°C conditions (Fig. 2H). If the morphotype difference between strains were producing a blanket transcriptional shift, these well-characterized Ire1-associated genes would also be affected — but they are not. This demonstrates selectivity in the Ire1-dependent response during morphogenesis.

Second, the morphogenesis cluster 6 genes show minimal overlap with established UPR gene sets from both *S. cerevisiae* (Travers et al. 2000; Supp Fig. 4E) and *C. albicans* (Yang et al. 2021; Supp Fig. 4F). If the morphogenesis cluster simply reflected a generic stress artifact of population heterogeneity, we would expect greater overlap with known stress-responsive gene sets.

Third, individual gene validations by RT-qPCR across multiple genetic backgrounds — including *hac1Δ/Δ*, which filaments at an intermediate level (~11%) — confirm the Ire1-dependent regulation of key targets (Fig. 6E, Fig. 7B). The *hac1Δ/Δ* strain provides a partial morphotype-matched control: it produces some filaments yet shows the same Ire1-dependent gene expression patterns as wild-type, arguing that the transcriptional changes reflect Ire1 signaling rather than morphotype composition.

We acknowledge that single-cell RNA-seq would in principle provide morphotype-resolved transcriptomes, but note that this approach remains technically challenging for filamentous *C. albicans* populations due to the difficulty of dissociating and capturing hyphal cells in standard droplet-based platforms.

2. The claim of regulated Ire1-dependent decay (RIDD) is currently unsupported by direct evidence of endonucleolytic activity. The data presented relies on steady-state abundance (RNA-seq/qPCR), which cannot distinguish between reduced synthesis and increased decay. To conclusively demonstrate RIDD, the authors must provide evidence of accelerated decay kinetics, map specific cleavage sites, or perform in vitro cleavage assays using the purified Ire1 cytosolic domain and potential RIDD substrates.

We performed two additional experiments to address this concern.

First, we utilized the transcriptional inhibitor thiolutin in combination with tunicamycin to monitor *ERG5* transcript decay kinetics in *ire1Δ/Δ+IRE1-WT* and *ire1Δ/Δ* strains (Supp Fig. 8A). *INO1* was used as a control UPR target gene (Supp Fig. 8B). While *ERG5* decay was modestly more pronounced in strains with functional Ire1, thiolutin treatment alone caused substantial *ERG5* decay and induced expression of *INO1*, suggesting that thiolutin itself activates stress responses that confound interpretation. This is consistent with recent findings that thiolutin has complex pleiotropic effects in vivo beyond transcription inhibition, including oxidative stress induction, Tor pathway inhibition, and metal homeostasis disruption (Qiu et al. 2024). These off-target effects made it impossible to cleanly isolate the Ire1-dependent decay component.

Second, we performed de novo motif discovery using STREME on the coding sequences of our

© 2026. Published by The Company of Biologists under the terms of the Creative Commons Attribution License (https://creativecommons.org/licenses/by/4.0/).

candidate genes, searching for enriched sequence elements that might represent Ire1 cleavage sites. While one of the most significant motifs (ACTGCTGCT; p=4.00E-06) contained a core GCTG element, Tomtom comparison revealed this corresponds to known transcription factor binding sites (SWI5/ACE2 in fungal databases) rather than a unique Ire1 recognition sequence. This is consistent with *C. glabrata*, where RIDD has been confirmed but no consensus motif has been identified (Miyazaki et al. 2013).

Based on these results, we have substantially revised our language throughout the manuscript. We no longer claim RIDD is present in *C. albicans*; instead, we describe "Ire1-dependent decreases in transcripts encoding secretory proteins" and note that the mechanism — whether direct endonucleolytic cleavage or indirect transcriptional regulation — remains to be determined. We discuss the limitation extensively in the Perspectives and Considerations section and suggest specific future experiments including in vitro cleavage assays with purified Ire1 RNase domain, Ski complex deletion mutants for detecting cleavage intermediates, and Northern blot analysis as described in Kimmig et al. (2012).

3. Critical phenotypes regarding the partial requirement of HAC1 and cell wall stress tolerance rely on deletion strains without matched genetic complementation. Without restoring gene function in the same background, it is impossible to rule out off-target edits or suppressor mutations. Complementation of the full deletion strains is standard practice and essential to validate causality, particularly given the reliance on these strains for the study's genetic arguments. Furthermore, introducing constitutively active spliced HAC1 into the ire1 deletion strain is necessary to fully test the independence of this pathway.

We have addressed this concern comprehensively with four new supplemental figures. As CRISPR-generated deletions in *C. albicans* are difficult to complement due to the consumption of selectable markers during biallelic disruption, we obtained independently generated and previously characterized *hac1Δ*, *hac1Δ+HAC1-WT*, *ire1Δ*, and *ire1Δ+IRE1-WT* strains constructed using conventional deletion and complementation approaches (Ramírez-Zavala et al. 2022). These strains provided essential matched genetic controls for validating phenotypes observed in our CRISPR deletion backgrounds.

Supp Fig. 6: We repeated the filamentation assay using these conventionally generated deletion/complement strains under multiple conditions (30°C untreated, 30°C serum, 37°C untreated, 37°C serum). The results confirm that *HAC1* deletion produces a significantly less severe filamentation defect than *IRE1* deletion, and that complementation rescues both phenotypes.

Supp Fig. 7: We introduced constitutively spliced *HAC1* (*HAC1**) into the *ire1Δ* background. *HAC1** rescued filamentation in *ire1Δ*, confirming that the Hac1-dependent component of the morphogenesis program can operate independently of Ire1 when *HAC1* is constitutively active. However, the rescue was incomplete compared to *ire1Δ+IRE1-WT*, consistent with additional Hac1-independent roles of Ire1 in morphogenesis.

Supp Fig. 10: The conventionally generated deletion/complement strains were tested on cell wall stressors (calcofluor white, caspofungin) and tunicamycin, confirming the differential requirement for *IRE1* versus *HAC1*. Cell wall gene expression (*IHD1*) was also validated in these strains (Supp Fig. 10B).

Supp Fig. 11: *HAC1** rescued growth of *ire1Δ* on cell wall stressors (calcofluor white, caspofungin), demonstrating that the cell wall stress sensitivity of *IRE1* deletion strains is at least partially attributable to loss of *HAC1* splicing. *HAC1* expression levels were validated by qPCR (Supp Fig. 11B), confirming elevated total *HAC1* in *HAC1** strains, consistent with the removal of intron-dependent translational repression and mRNA degradation mechanisms (Matabishi-Bibi et al. 2022; Cox and Walter 1996).

Together, these experiments validate all key phenotypes in independently generated strains with matched genetic controls and directly test the Hac1-dependence of the *ire1Δ* phenotypes using constitutively active *HAC1*.

© 2026. Published by The Company of Biologists under the terms of the Creative Commons Attribution License (https://creativecommons.org/licenses/by/4.0/).

Reviewer 1 — Minor Points

4. The comparison between the canonical UPR (tunicamycin at 30°C) and the morphogenesis program (serum at 37°C) is confounded by differences in temperature and duration. These variables alone can shift gene expression, artificially inflating the apparent divergence between the programs. Temperature-matched controls or time-course analyses are needed to confirm that the "distinctness" is not an artifact of experimental conditions.

We acknowledge this potential confound and have addressed it in the Discussion section ("Hac1-independent regulation of hyphal growth by Ire1"). While the different experimental conditions were necessary to elicit the relevant biological responses (tunicamycin causes ER stress optimally at 30°C; serum-induced filamentation requires 37°C), several lines of evidence argue that the observed transcriptional divergence reflects genuine program distinctness rather than temperature/duration artifacts. RT-qPCR validation confirmed that canonical UPR targets (*KAR2*, *PDI1*) were not induced by serum+37°C and the morphogenesis gene *AHP1* was not induced by tunicamycin (Supp Fig. 4A-C), demonstrating condition-specific regulation. Furthermore, the morphogenesis cluster showed minimal overlap with published UPR gene sets from both *S. cerevisiae* and *C. albicans* (Supp Fig. 4E,F). If the divergence were driven primarily by temperature or duration rather than fundamentally different Ire1 signaling programs, we would expect greater overlap between the two gene sets and less condition-specific regulation of individual targets.

5. The conclusion that morphogenesis occurs without HAC1 splicing relies on a gel-based assay that lacks sufficient sensitivity to detect low-amplitude or transient events. As splicing can be subtle during physiological transitions, a more sensitive quantitative method, such as qPCR, is required to definitively rule out minor splicing events.

We designed RT-qPCR primers to separately detect spliced and total *HAC1*, providing substantially greater sensitivity than the gel-based assay. This approach revealed minor but statistically significant increases in spliced *HAC1* at 30 and 60 minutes post-serum+37°C treatment, which disappeared by 120 minutes (Fig. 5E). In contrast, tunicamycin treatment produced robust and sustained *HAC1* splicing. We have revised the section title to "Limited *HAC1* splicing occurs during morphogenesis" and adjusted the text throughout to reflect this finding. This result is highly informative: the transient, low-level *HAC1* splicing during early morphogenesis is consistent with a threshold model in which morphogenesis-associated signals activate Ire1 to a level that permits limited *HAC1* splicing and engagement of alternative outputs, but falls short of the sustained activation triggered by proteotoxic stress. We discuss this model in the new "Differential Ire1 signaling and perspectives" section, drawing on recent mechanistic studies showing that *HAC1/XBP1* splicing and RIDD represent qualitatively distinct catalytic modes of Ire1 with different oligomerization requirements (Tam et al. 2014; Bright et al. 2015; Le Thomas et al. 2021).

6. The use of a hypomorphic IRE1 strain for the transcriptomic inference of Ire1 dependence may conflate residual signaling or secondary stress effects with specific Ire1-dependent regulation. Validating key targets using clean deletion and rescue strains in the same experimental design would strengthen the attribution of these gene sets to Ire1 function.

We acknowledge that CRISPR-generated deletion strains in *C. albicans* are notoriously difficult to complement due to the lack of convenient selectable markers after both alleles have been disrupted. To address this concern, we obtained independently generated and previously characterized *ire1Δ*, *hac1Δ*, and corresponding complemented strains constructed using conventional gene deletion and reintegration approaches (Ramírez-Zavala et al. 2022). These strains allowed us to validate all key phenotypes — filamentation (Supp Fig. 6), cell wall stress sensitivity (Supp Fig. 10), and cell wall gene expression (Supp Fig. 10B) — in deletion/complement pairs, confirming that the results obtained with our CRISPR *ire1Δ/Δ* and *hac1Δ/Δ* strains and the *ire1DX* hypomorphic strain reflect genuine Ire1- and Hac1-dependent regulation. Additionally, the RT-qPCR validations of candidate secretory transcript targets (Fig. 6E) and cell wall genes (Fig. 7B) were performed across multiple strain backgrounds, providing independent confirmation of Ire1-dependent regulation.

© 2026. Published by The Company of Biologists under the terms of the Creative Commons Attribution License (https://creativecommons.org/licenses/by/4.0/).

Reviewer 2

(1) As the authors describe in the manuscript, some previous publications have already suggested Hac1-independent roles of Ire1 in Candida cells. I think that conceptual novelty and importance of the present study should be explained more understandably and clearly to highlight differences from the previous studies.

We have added a paragraph in the Discussion section ("Hac1-independent regulation of hyphal growth by Ire1") that explicitly articulates three ways our study extends prior observations. First, previous studies established that Ire1 and Hac1 are separable in specific phenotypes (iron uptake, manganese tolerance, colony morphology); our genome-wide transcriptomic analysis reveals that the Ire1-dependent programs during proteotoxic stress and morphogenesis are fundamentally distinct, with only 24 of over 800 Ire1-upregulated genes shared between conditions. Second, we provide the first systematic identification of Ire1-dependent, Hac1-independent decreases in transcripts encoding secretory pathway proteins in *C. albicans* — a pattern not previously reported in this organism. Third, we establish cell wall integrity as a specific Hac1-independent output of Ire1 during morphogenesis through genetic dissection with multiple strain backgrounds. We have also added a sentence to the Introduction clarifying that the specific Ire1-dependent transcriptional programs driving morphogenesis had not been defined at a genome-wide level prior to this study.

(2) According to the authors' model shown in Fig. 8, the control of cell wall integrity and the RIDD are two distinct outputs from Ire1. Please explain why authors think so. Alternatively, is it possible that, for instance, cell wall integrity is controlled as a downstream event of the RIDD?

This is an excellent point. We have revised Figure 8 to reflect the possibility that these outputs may be mechanistically linked rather than independent. We have also added a new paragraph in the Discussion ("Cell wall integrity and morphogenesis") that directly addresses this question. Given that many of the Ire1-dependent downregulated transcripts encode cell surface and cell wall proteins (Fig. 6C,D), selective degradation of these mRNAs could directly remodel the cell wall proteome during the yeast-to-hyphal transition. Conversely, the simultaneous Ire1-dependent upregulation of different cell wall biosynthesis genes (Fig. 7B) suggests a parallel transcriptional program operating alongside any post-transcriptional regulation. We note that distinguishing between these models will require targeted disruption of individual candidate genes to determine whether their degradation is functionally required for cell wall remodeling during morphogenesis.

(3) In the Discussion section, please more clearly explain possible mechanism(s) by which Ire1 causes different outputs in response to tunicamycin and to serum+37C. By what mechanism did Ire1 fail to promote HAC1-mRNA splicing when cells were exposed to serum+37C? By what mechanism did Ire1 fail to promote gene expression changes leading to hyphal formation when cells were stressed by tunicamycin?

We have added a substantial new Discussion section titled "Differential Ire1 signaling and perspectives" that addresses this central question with reference to recent mechanistic advances in the field. We discuss three key findings from mammalian systems that provide a framework for understanding our observations:

First, *XBP1*/*HAC1* splicing and RIDD require fundamentally different Ire1 oligomeric states — cooperative oligomers for *HAC1* splicing versus non-cooperative dimers for RIDD (Tam et al. 2014). This provides a molecular basis for how Ire1 could engage in alternative outputs without robust *HAC1* splicing.

Second, RIDD cleavage is temporally separate from *XBP1* splicing and occurs under specific activation conditions (Bright et al. 2015), establishing that these are not simply graded versions of the same activity.

© 2026. Published by The Company of Biologists under the terms of the Creative Commons Attribution License (https://creativecommons.org/licenses/by/4.0/).

Third, mammalian IRE1α possesses multiple RIDD modalities including one (RIDDLE) that does not require consensus sequence recognition (Le Thomas et al. 2021), and the relationship between *HAC1/XBP1* splicing and RIDD varies dramatically across evolution — from *S. cerevisiae* (HAC1 splicing only) to *S. pombe* (RIDD only) to mammals (both). We suggest that *C. albicans* may occupy a position in this spectrum more similar to mammalian cells, retaining both outputs, though the extent to which mammalian mechanisms are conserved in pathogenic fungi remains to be determined.

We propose a threshold model in which morphogenesis-associated signals activate Ire1 to a level sufficient for limited *HAC1* splicing and alternative outputs but insufficient for the sustained, high-level activation triggered by proteotoxic stress. The transient *HAC1* splicing we detected at early timepoints during serum+37°C treatment (Fig. 5E) is consistent with this model.

(4) In the manuscript, it is not clear whether the KD mutant Ire1 has an RNase activity as strong as wild-type Ire1. Autophosphorylation of wild-type Ire1 is a prerequisite for its activation as an RNase. Did the KD mutant exhibit diminished functions simply because its RNase activity was impaired by the KD mutation? Alternatively, can the authors present evidence to claim a role of Ire1's kinase other than autophosphorylation?

We have addressed this in the Perspectives and Considerations section. We acknowledge that the kinase-dead mutation could impair filamentation indirectly by reducing endoribonuclease activation rather than through a distinct kinase function. While Ire1 autophosphorylation is classically considered a prerequisite for RNase activation, studies in *S. cerevisiae* have shown that kinase autophosphorylation is actually dispensable for endoribonuclease activation, although it is required for subsequent Ire1 deactivation (Rubio et al. 2011; Chawla et al. 2011; Han et al. 2009). This raises the possibility that the kinase-dead phenotype reflects a failure to properly regulate the duration or dynamics of Ire1 signaling rather than simply a loss of RNase activity. We do not currently have evidence that yeast Ire1 phosphorylates substrates other than itself, and we state that further studies are needed to definitively separate the kinase and RNase functions of Ire1 in the context of morphogenesis.

(5) As shown in Kimmig et al. (PMID: 23066505), short fragments of downregulated RNA produced dependently on Ire1 are strong evidence of RIDD. Therefore, I think that a limitation of this study is the lack of data to demonstrate that the downregulated genes shown in Fig 7 are really digested by Ire1. Please address this point in the Discussion section.

We fully agree that this is a significant limitation and have addressed it in multiple sections of the revised manuscript. In the Results, we present the thiolutin transcriptional inhibition experiments and motif analysis as honest attempts to probe the mechanism, noting that both approaches were inconclusive — the former due to pleiotropic effects of thiolutin (Supp Fig. 8), and the latter because enriched motifs matched known transcription factor binding sites rather than unique Ire1 recognition elements. In the Discussion, we acknowledge that our data are consistent with but do not confirm RIDD in *C. albicans*, and we have systematically replaced definitive RIDD language with more cautious phrasing ("Ire1-dependent reduction in transcripts encoding secretory proteins"). We have also added a dedicated paragraph in Perspectives and Considerations outlining three specific experimental approaches that could resolve this question: in vitro cleavage assays with purified Ire1 RNase domain (Lee et al. 2011), Northern blot analysis in Ski complex deletion mutants to detect Ire1-dependent cleavage intermediates (Brown et al. 2000; Kimmig et al. 2012), and mapping of cleavage sites. We note that the absence of a consensus motif is not in itself inconsistent with RIDD activity, as *C. glabrata* RIDD targets also lack a consensus recognition sequence (Miyazaki et al. 2013), and mammalian IRE1α possesses a non-canonical RIDDLE mode that cleaves substrates without endomotif recognition (Le Thomas et al. 2021).

Second decision letter

MS ID#: jcs.264610R1

© 2026. Published by The Company of Biologists under the terms of the Creative Commons Attribution License (https://creativecommons.org/licenses/by/4.0/).

MS Title: Hac1-independent functions of Ire1 drive morphogenesis in Candida albicans through distinct transcriptional programs

Authors: Samuel Stack-Couture; Gabriela Librais; Bryan Lung; Julie Genereaux; Viola Halder; Vanessa Dumeaux; Rebecca Shapiro; Patrick Lajoie

Article Type: Research Article

Dear Dr Lajoie,

I am happy to tell you that your manuscript has been accepted for publication in Journal of Cell Science, pending standard publication integrity checks and a few minor textual changes suggested by reviewer 1.

Reviewer 1

The authors have addressed my concerns thoroughly. The new complementation strains, the reframing of the RIDD claims, and the handling of the morphotype confound resolve the substantive issues. One point should be clarified before publication.

Remaining points
* The manuscript frames cell wall integrity as Hac1-independent, yet Supp Fig 11 shows HAC1* rescues ire1Î" growth on cell wall stressors. The authors should separate the two components. Cell wall stress tolerance is partly Hac1-dependent, whereas upregulation of GPI-anchored cell wall genes (Fig 7B) is Hac1-independent.
* The HAC1* strains show large increases in total HAC1 (Supp Fig 11B). The rescue may therefore reflect overexpression rather than constitutive splicing alone. A sentence acknowledging this caveat would suffice.

Reviewer 2

The manuscript has been revised well. It can now be accepted for publication in Journal of Cell Science.

© 2026. Published by The Company of Biologists under the terms of the Creative Commons Attribution License (https://creativecommons.org/licenses/by/4.0/).
